# The Global Kidney Patient Trials Network and the CAPTIVATE Platform Clinical Trial Design: A Trial Protocol

**DOI:** 10.1001/jamanetworkopen.2024.49998

**Published:** 2024-12-11

**Authors:** Sradha S. Kotwal, Vlado Perkovic, Meg J. Jardine, Dana Kim, Nasir A. Shah, Enmoore Lin, Sarah Coggan, Laurent Billot, Priya Vart, David C. Wheeler, Ian H. de Boer, Hong Zhang, Fan Fan Hou, Yuka Sugawara, Joseph Marion, Roger J. Lewis, Lindsay R. Berry, Anna McGlothlin, Vivekanand Jha, Luca De Nicola, Jose L. Gorriz, Hiddo J. L. Heerspink

**Affiliations:** 1 Renal and Metabolic Division, The George Institute for Global Health, University of New South Wales, Sydney, Australia; 2 Prince of Wales Hospital, Sydney, New South Wales, Australia; 3 Faculty of Medicine, University of New South Wales, Sydney, Australia; 4 National Health and Medical Research Council Clinical Trials Centre, Sydney, Australia; 5 The University of Sydney, Sydney, Australia; 6 Department of Renal Medicine, Concord Repatriation General Hospital, Sydney, Australia; 7 Department of Clinical Pharmacy and Pharmacology, University of Groningen, Groningen, the Netherlands; 8 University Medical Center Groningen, Groningen, the Netherlands; 9 University College London Centre for Kidney and Bladder Health, London, United Kingdom; 10 Kidney Research Institute, University of Washington, Seattle; 11 Renal Division, Peking University First Hospital, Beijing, China; 12 Division of Nephrology, Nanfang Hospital, Southern Medical University, Guangzhou, China; 13 National Clinical Research Center for Kidney Disease, Guangzhou, China; 14 Department of Nephrology and Endocrinology, The University of Tokyo Hospital, Tokyo, Japan; 15 Berry Consultants LLC, Austin, Texas; 16 Department of Emergency Medicine, David Geffen School of Medicine at University of California, Los Angeles; 17 The George Institute for Global Health, University of New South Wales, New Delhi, India; 18 School of Public Health, Imperial College, London, United Kingdom; 19 Prasanna School of Public Health, Manipal Academy of Higher Education, Manipal, India; 20 Nephrology and Dialysis Unit, Department of Advanced Medical and Surgical Sciences, University Vanvitelli, Naples, Italy; 21 Department of Nephrology, Hospital Clínico Universitario de Valencia, Universitat de València, Valencia, Spain

## Abstract

**Importance:**

Chronic kidney disease (CKD) is a global health priority affecting almost 1 billion people. New therapeutic options and clinical trial innovations such as adaptive platform trials provide an opportunity to efficiently test combination therapies.

**Objective:**

To describe the design and baseline results of the Global Kidney Patient Trials Network (GKPTN) and the design and structure of the global adaptive platform clinical trial Chronic Kidney Disease Adaptive Platform Trial Investigating Various Agents for Therapeutic Effect (CAPTIVATE) to find new therapeutic options and treatments for people with kidney disease.

**Design, Setting, and Participants:**

The GKPTN is a multicenter registry that started in May 2020 and is ongoing, while CAPTIVATE is a multicenter, multifactorial, phase 3, placebo-controlled adaptive platform randomized clinical trial that includes patients with CKD. The first participant was randomized in September 2024. The GKPTN recruits patients from kidney and endocrinology practices, and CAPTIVATE aims to recruit patients from GKPTN sites where possible. Both the GKPTN and CAPTIVATE recruit patients with nondialysis CKD.

**Intervention:**

CAPTIVATE will test several investigational agents or combinations of agents, beginning with a mineralocorticoid receptor antagonist.

**Main Outcomes and Measures:**

The GKPTN monitors clinical characteristics, treatment, and outcomes to identify eligible clinical trial participants and provide a contemporary global picture of patients with CKD. The primary outcome of CAPTIVATE is to identify investigational agents or combinations of agents to reduce the rate of chronic estimated glomerular filtration rate (eGFR) decline. The default maximum sample size per treatment arm in each domain, based on bayesian simulations, is 500 participants, providing approximately 90% power to detect a clinically meaningful improvement of 2.6 mL/min/1.73 m^2^ in eGFR at the end of the 104-week study period.

**Results:**

The GKPTN has enrolled 4334 patients across 119 sites in 8 countries (US, Australia, Argentina, China, Italy, Canada, Spain, and Japan). The mean (SD) participant age at enrollment was 64.5 (16.2) years, 2542 participants (58.7%) were female, and diabetic kidney disease was most frequently reported among patients for CKD etiology (1875 [43.3%]). Among the participants, the mean (SD) eGFR was 52.9 (29.3) mL/min/1.73 m^2^, and the median urinary albumin-to-creatinine ratio was 89 mg/g (coefficient of variation, 20-420 mg/g). In the GKPTN cohort, the mean eGFR decline was steeper among participants with a baseline eGFR of 60 mL/min/1.73 m^2^ or more (−2.29 [95% CI, −3.14 to −1.44]) compared with those with an eGFR of less than 60 mL/min/1.73 m^2^ (−1.16 [95% CI, −1.77 to −1.44]) and was progressively steeper in more severe albuminuria subgroups.

**Conclusions and Relevance:**

The GKPTN registry and the CAPTIVATE trial have the potential to expand and optimize therapeutic options for people with CKD using an adaptive platform clinical trial design.

**Trial Registration:**

ClinicalTrials.gov Identifiers: NCT04389827 and NCT06058585

## Key Points

**Question:**

How can adaptive platform trials be designed for nephrology?

**Findings:**

The Global Kidney Patient Trials Network (GKPTN) registry has enrolled 4334 patients with chronic kidney disease since May 2020 across 119 sites in 8 countries. The Chronic Kidney Disease Adaptive Platform Trial Investigating Various Agents for Therapeutic Effect (CAPTIVATE) is a platform randomized clinical trial that includes patients with chronic kidney disease and intends to identify new therapeutic options and clinical trial innovations to efficiently test combination therapies using bayesian approaches and trial simulations to estimate sample size.

**Meaning:**

The GKPTN registry and the CAPTIVATE trial could find new treatments for people with kidney disease and change how trials are conducted in patients with kidney disease.

## Teaser

This trial protocol identifies the design and baseline results of the Global Kidney Patient Trials Network (GKPTN) and the global adaptive platform randomized clinical trial Chronic Kidney Disease Adaptive Platform Trial Investigating Various Agents for Therapeutic Effect (CAPTIVATE) to find new therapeutic options and treatments for people with kidney disease.

## Introduction

Chronic kidney disease (CKD) is a global health priority that affects almost 1 billion people and is predicted to rise to the fifth-highest cause of death in 2040.^1^ The increasing global prevalences of obesity, type 2 diabetes, and hypertension are contributing factors for the continuous surge in the number of people with CKD. Approximately 40% of patients with type 2 diabetes develop CKD during their lifetime.^2,3^ Patients with CKD experience a disproportionately higher risk of cardiovascular events.^4^ Effective treatments to reduce the risk of kidney and cardiovascular complications are thus highly desired.

Nephrology has traditionally lagged behind other medical disciplines in the completion of randomized clinical trials to guide therapy.^5^ High costs, lack of global clinical trial networks, and a limited number of known and validated mechanisms of disease have all been identified as contributing factors.^6^ During the past decade, the tide has been turning with identification of new therapies such as sodium-glucose cotransporter-2 inhibitors, the nonsteroidal mineralocorticoid receptor antagonist finerenone, the endothelin receptor antagonist sparsentan, and the glucagon-like peptide-1 receptor agonist semaglutide, which are all proven to be effective in reducing albuminuria (an accepted surrogate outcome in CKD clinical trials) and/or clinical kidney end points in patients with CKD, with and without type 2 diabetes.^7,8,9,10,11,12^

The growing number of randomized clinical trials testing the efficacy and safety of other novel interventions poses both opportunities and challenges. An increasing number of proven, effective interventions necessitate defining optimal therapeutic combinations and personalization of therapies (single or multiple).^13^ While new therapeutics improve outcomes, they do not entirely ameliorate the risk, highlighting the need for further innovation to address these gaps. Increased trial numbers also strain patient and site resources, potentially lengthening the time needed to test new (combination) therapies.

Innovative trial designs offer an opportunity to address these challenges by reducing the time taken to test new therapies, establishing infrastructure to add new interventions in an efficient fashion, and therefore allowing the identification of optimal therapeutic combinations for patients in a timely fashion. An adaptive platform trial is an innovation in clinical trial design and methodology that allows the evaluation of multiple interventions in a single disease, or a group of related diseases, within a standing single trial infrastructure, often with no fixed end date or maximum sample size.^14^ Adaptive platform trials are well established in oncology^15,16^ and infectious disease.^17,18^ Recent advances in trial designs, statistical techniques, acceptance of surrogate end points for kidney disease, and the ability to use clinical trial enrichment approaches to identify people at high risk, together with the identification of new interventions targeting distinct disease pathways, have paved the way for the introduction of innovative trial designs within nephrology such as adaptive platform trials.^19,20,21^ This scientific revolution has the potential to cause a paradigm shift in the way future clinical trials are conducted and how therapies are developed and tested for people with CKD.^22^

To meet the purpose of developing a platform trial in nephrology, the first step is to establish a large network of patients with various causes of CKD who are interested in participating in clinical trials and to find sites that are interested in enrolling patients into clinical trials. The Global Kidney Patient Trials Network (GKPTN) registry was designed to address this. The second step is to develop a master protocol that accommodates a variety of clinical trials to test multiple interventions and uses adaptive designs and bayesian statistical comparisons. The Chronic Kidney Disease Adaptive Platform Trial Investigating Various Agents for Therapeutic Effect (CAPTIVATE) platform trial leverages this opportunity to improve the current clinical trial framework in terms of time, efficiency, and costs.

This study describes the design and characteristics of the GKPTN registry and participants and the outline of the study design for the CAPTIVATE platform trial. The CAPTIVATE platform trial by default will recruit from within the GKPTN, although participation of non-GKPTN clinical practice sites can be considered.

## Methods

### Design of the GKPTN

#### Study Design

The GKPTN registry, an ongoing multicenter registry of patients with CKD that started in May 2020, aims to identify potentially eligible participants for interventional clinical trials and to monitor their long-term outcomes. Adhering to the Declaration of Helsinki^23^ and the International Conference on Harmonization Guidelines for Good Clinical Practice,^24^ the study protocol received ethical approval in all participating countries from all participating institutions. Written informed consent, including willingness for future trial participation, was obtained from all participants before any study procedures. This registry follows the Standard Protocol Items: Recommendations for Interventional Trials (SPIRIT) reporting guideline.

#### GKPTN Participants

All patients with incident and prevalent CKD at participating centers are eligible for inclusion (criteria are detailed in eTable 1 in Supplement 1). Potential patients are identified by site staff using medical records.

#### GKPTN Data Collection and Follow-Up

Deidentified data are collected from the patients’ medical records and entered electronically into study-specific electronic case report forms, housed within a bespoke database. Baseline demographic data including age, sex, cause of kidney disease, current treatments, and other relevant medical conditions and pathology data including estimated glomerular filtration rate (eGFR), albuminuria, and cardiac biomarkers are collected from patient records by site staff at the time of recruitment. Race and ethnicity, defined as recorded in the medical records (by physicians or self-report across jurisdictions), are collected because there are known differences in rates, types, and progression of kidney disease across different ethnicities. Categories, as determined by the investigators, were American Indian or Alaska Native, Oceanian (including Aboriginal people, Māori people, Native Hawaiian or Other Pacific Islander individuals, or Torres Strait Islander individuals), or other (data did not specify categories); Asian; Black or African American; Hispanic or Latino; and White. Follow-up data, including eGFR, albuminuria, and clinical kidney and cardiovascular outcomes, are collected from medical records during routine clinical visits. All data are stored according to local regulatory requirements and accessed only by approved study personnel.

#### Statistical Methods

Data entered by site staff in the electronic case report forms are regularly checked for validity and accuracy. Descriptive statistics are used to describe the characteristics of recruited participants. eGFR trajectories and the annual rate of eGFR decline are estimated from linear mixed-effects models.

### Design of the CAPTIVATE Platform Trial

#### Study Design

CAPTIVATE is a multicenter, multifactorial, phase 3, placebo-controlled, parallel group, adaptive platform randomized clinical trial that includes patients with CKD. The first participant was randomized in September 2024. CAPTIVATE adheres to the Declaration of Helsinki^23^ and the International Conference on Harmonization Guidelines for Good Clinical Practice.^24^ The study protocol, domain-specific appendices (DSAs), amendments, and consent forms undergo ethical review and approval by health authorities and independent review boards and ethics committees according to country-specific requirements. All participants will provide written informed consent or electronic consent before any study procedures. The study will follow the Standard Protocol Items: Recommendations for Interventional Trials (SPIRIT) reporting guideline.

The CAPTIVATE platform trial uses a modular protocol design that allows the trial to efficiently evolve over time. The primary objective is to determine investigational agents or combinations of agents that slow progression of CKD, compared with placebo, in patients with CKD receiving standard-of-care therapy with a variety of therapeutic agents planned to be studied.

**Figure 1.**
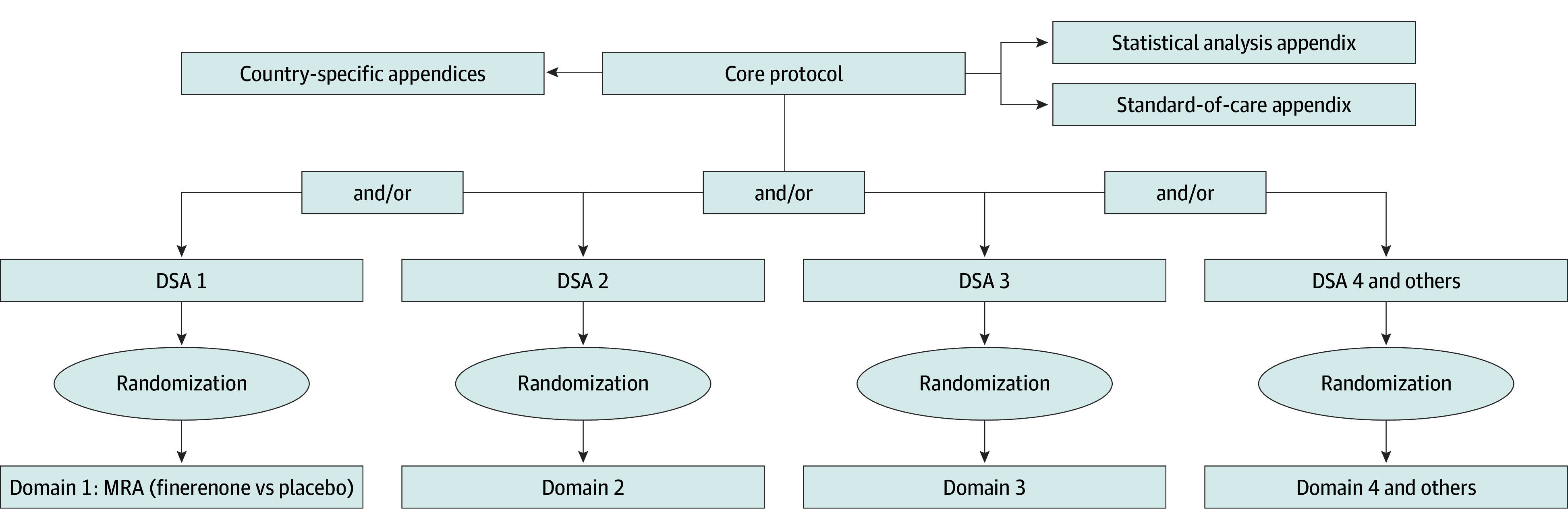
The Chronic Kidney Disease Adaptive Platform Trial Investigating Various Agents for Therapeutic Effect (CAPTIVATE) Study Design DSA indicates domain-specific appendix; MRA, mineralocorticoid receptor antagonist.

The assessment of therapeutic strategies is organized into domains, which consist of multiple mutually exclusive treatment options (ie, interventions). Participants can be randomized into any active domain for which they are eligible (multifactorial randomization) and can also be enrolled in additional domains sequentially as new domains are commenced. The CAPTIVATE protocol consists of a core protocol, multiple DSAs, multiple country-specific appendices, a statistical analysis appendix, and a standard-of-care appendix (Figure 1). This design allows interventions and countries to be introduced or removed without altering the core protocol. Trial features, procedures or ethical considerations specific to a particular domain, or country or region are outlined separately in the respective appendices.

##### Core Protocol

The core protocol (available in Supplement 2) contains information that applies to all domains and countries involved in the platform trial. The information includes the trial background, design, procedures, outcomes (efficacy and safety), principles of statistical analyses, trial governance, and ethical considerations.

##### Participants

Adults who are 18 years or older with known CKD from any cause with an eGFR of 25 mL/min/1.73 m^2^ or more, eligible for randomization in at least 1 recruiting DSA, and willing and able to provide written or electronic informed consent and to comply with all study visits and study procedures are eligible for inclusion. DSA-specific criteria could include markers of risk such as levels of albuminuria or presence of comorbidity. Key exclusion criteria for the core protocol are current maintenance dialysis treatment, planned kidney transplantation within 6 months of inclusion in a DSA, or a life expectancy of less than 6 months. Domain-specific inclusion and exclusion criteria will be outlined in each respective DSA.

##### DSAs

The DSA details domain-specific interventions integrated into CAPTIVATE, supplementing the core protocol with additional characteristics or design features. These may include but are not limited to narrower inclusion or exclusion criteria, DSA-specific outcomes, varied randomization strategies (eg, fixed randomization or response-adaptive randomization), sample size, success-stopping or futility-stopping criteria, and rescue randomization for nonresponders.^25^

##### Interventions

Interventions include treatments showing potential benefit in CKD. The platform oversight committee—including investigators, statisticians, key opinion leaders, and consumers—approves new interventions to CAPTIVATE based on prespecified criteria, which include a strong supporting hypothesis, potential for clinical translations, feasibility of integration into CAPTIVATE, recruitment capacity, and funding availability.

The CAPTIVATE trial will begin recruitment with the mineralocorticoid receptor antagonist domain in quarter 3 of 2024, with further domains planned to be added in 2025. Domain-specific details will be published separately.

Intervention details are outlined in the DSAs. Each domain is overseen by a domain steering committee of investigators, intervention experts, and regional leaders who will evaluate DSA suitability for specific locations. All available DSAs in a region are offered to each site. The modular design enables each site to choose the interventions within CAPTIVATE in which it wishes to participate.

##### Follow-Up Assessments

In-person study visits are scheduled at prespecified intervals until 104 weeks for each domain, with a final study visit at 108 weeks. After 104 weeks’ treatment, study intervention will be discontinued, and participants will proceed into a 4-week washout period to assess off-treatment effects. The trial aims to align all study visits (core-specific and domain-specific visits) to occur concurrently with clinical care to minimize patient and site burden.

Serum creatinine will be assessed at all in-person study visits to enable calculation of eGFR (in milliliters per minute per 1.73 square meters) using the 2021 Chronic Kidney Disease Epidemiology Collaboration equation,^26^ based on creatinine, age, and sex using local laboratories. First-morning void urine samples will be collected at baseline and all follow-up visits for assessment of urinary albumin and creatinine using local laboratory measurements.

##### Outcomes

The default within CAPTIVATE is that all outcomes in the core protocol will apply to each DSA, with any departure from the core outcomes detailed in the relevant DSA. The core protocol outcomes will be collected for all patients enrolled within the CAPTIVATE platform.

The primary outcome is the chronic eGFR slope measured from week 4 to the end-of-treatment visit at week 104 (eTable 2 in Supplement 1). The key secondary outcomes are the change from baseline in albuminuria at week 24, the change in eGFR from randomization to the end of washout, and the proportion of participants experiencing a 40% eGFR decline or developing kidney failure (defined as eGFR <15 mL/min/1.73 m^2^ or chronic kidney replacement therapy start) at 108 weeks. Other secondary exploratory outcomes and outcomes of special interest are described in eTable 2 in Supplement 1. Safety will be assessed through investigator-reported and adjudicated adverse events, with intervention-specific safety outcomes described in DSAs.

### Statistical Analysis

A result will be obtained and reported from each intervention tested in a domain using a single integrated analysis, and a detailed statistical analysis plan will be developed and published prior to the completion of each domain. Broadly, using an intention-to-treat approach, the primary analysis population includes all patients who are eligible for the treatment arm of interest and concurrently randomized. The primary outcome of the chronic eGFR slope from week 4 to week 104 will be estimated using a bayesian mixed model for repeated measures for each intervention relative to placebo, measured from the time of randomization to the end of washout. A posterior probability greater than 0.985 is needed for success; this adjusted threshold accounts for repeated testing at interim analyses. The secondary end point of change in the urinary albumin-to-creatinine ratio (UACR) will also be analyzed with a mixed model for repeated measures and the time-to-event end points using Cox proportional hazards regression models. By default, each primary analysis will assume no interactions, and postconclusions’ secondary analyses will further assess this assumption. However, if an interaction between interventions is deemed plausible and potentially large, the primary analyses for those interventions may be modified to incorporate an interaction term.

#### Approach to Interim Analyses

**Figure 2.**
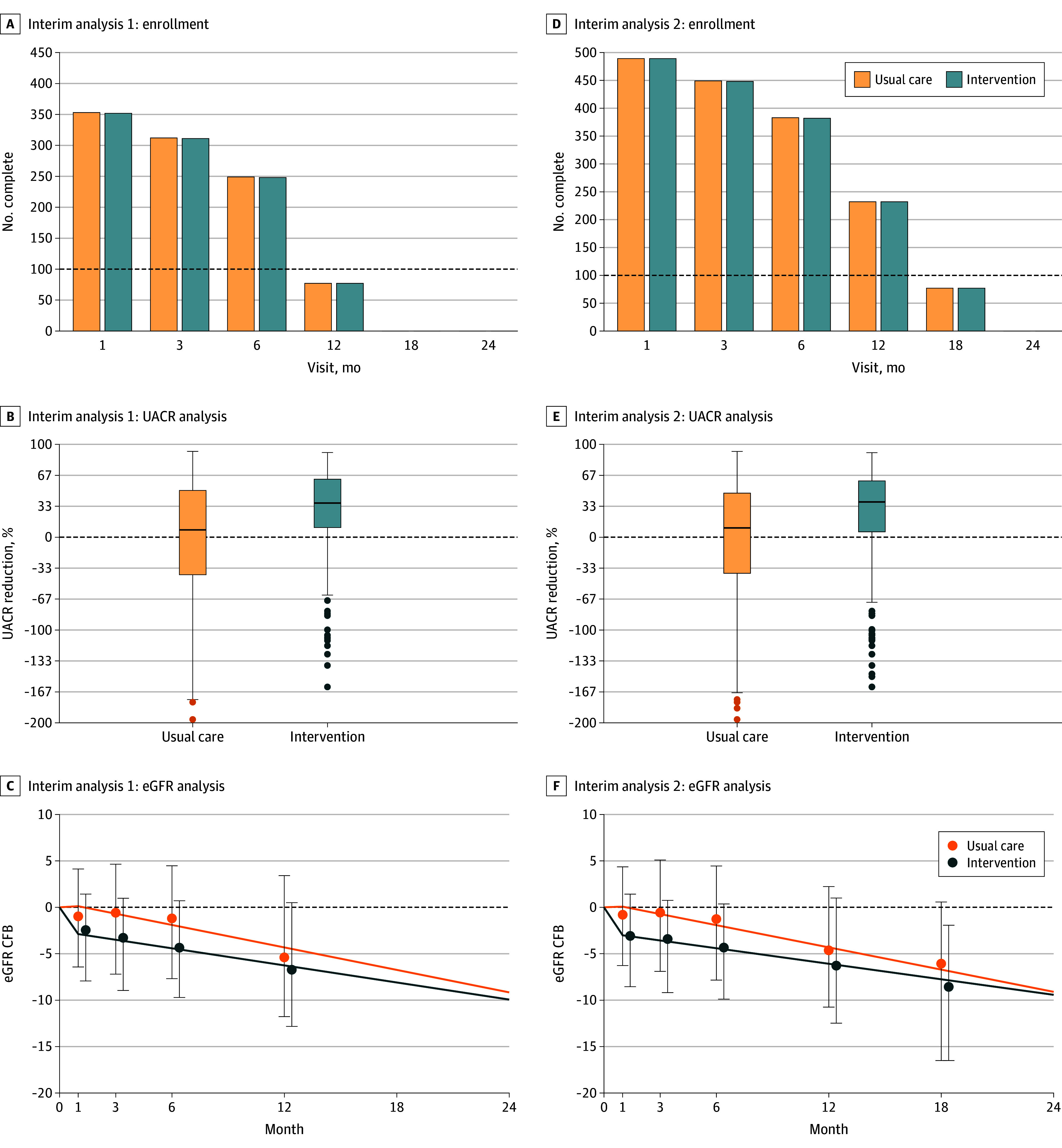
Interim Analyses for 1 Domain Within the CAPTIVATE Platform After 18 and 24 Months Interim analyses for a 2-arm domain with the default-stopping rules for a single simulated example trial are shown. A-C, Interim analysis 1 is performed 18 months after the domain is open. A total of 745 participants have been randomized within the domain, and at least 100 participants per treatment arm have completed the 6-month visit. The intervention is assessed for futility based on the urinary albumin-to-creatinine ratio (UACR) end point. The treatment effect is estimated to reduce UACR by 31.2% (95% CI, 21.9%-39.3%), and the probability of providing a clinically important 25% reduction is 0.909; therefore, futility is not declared for the intervention, and the trial continues to recruit. While there is some preliminary data suggesting that the treatment also reduces the eGFR slope (difference in eGFR slope, 1.11 [95% CI, −1.73 to 3.95] mL/min/1.73 m^2^), there is insufficient follow-up to assess either success or futility on this end point. The probability of a slope effect greater than 0 mL/min/1.73 m^2^ per year is 0.779; the probability of a slope effect greater than 0.8 mL/min/1.73 m^2^ per year is 0.586. D-F, Interim analysis 2 is performed at 24 months. Enrollment is complete, and the domain has reached the maximum sample size (N = 1000). The treatment continues to show a strong benefit on the UACR and does not meet the futility criteria. The treatment effect is estimated to reduce UACR by 28.4% (95% CI, 21.2%-35.0%), and the probability of providing a clinically important 25% reduction is 0.830. The intervention is eligible for futility stopping on the eGFR end point, but the probability of providing a clinically important 0.8-mL/min/1.73 m^2^ per year difference compared with placebo in the eGFR slope is 0.778; therefore, the intervention is not stopped for futility, and the trial continues to recruit. The difference in the eGFR slope is 1.38 (95% CI, −0.11 to 2.88) mL/min/1.73 m^2^. The probability of a slope effect greater than 0 mL/min/1.73 m^2^ per year is 0.965. Error bars indicate 95% CIs. In panels B and E, horizontal lines indicate the median change in UACR from baseline, and boxes indicate the IQR; the dots indicate outliers. CFB indicates change from baseline.

**Figure 3.**
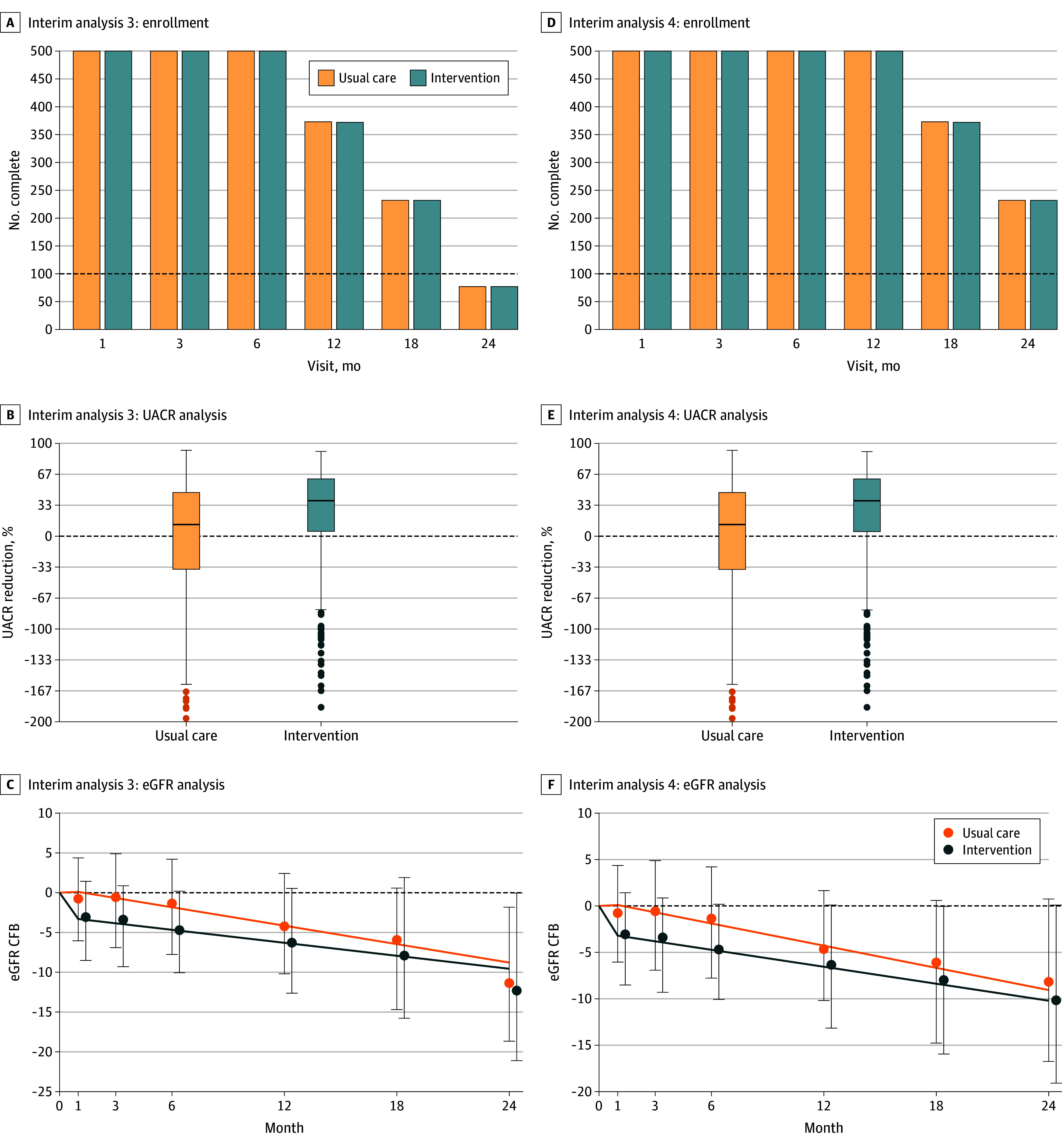
Interim Analyses for 1 Domain Within the CAPTIVATE Platform After 30 and 36 Months Two interim analyses after 30 and 36 months’ follow-up for domain-specific appendices, in which follow-up continues after the 24-month interim analysis described in Figure 2, are shown. A-C, For interim analysis 3, which occurs after 30 months’ follow-up, a total of 1000 participants have been randomized, and the treatment continues to show a strong benefit on the urinary albumin-to-creatinine ratio (UACR) (UACR reduction, 29.1% [95% CI, 22.7%-34.9%]; probability of providing a clinically important 25% reduction, 0.899) and the eGFR slope (1.32 [95% CI, 0.24-2.39] mL/min/1.73 m^2^ per year). While the posterior probability of superiority on the eGFR is above the 0.985 threshold (probability of slope effect >0 mL/min/1.73 m^2^ per year, 0.992), the number of participants assigned to the intervention with a complete 108-week follow-up is less than 100; thus, the intervention is not eligible to stop for success, and the trial continues. The probability of a slope effect greater than 0.8 mL/min/1.73 m^2^ per year is 0.826. D-F, For interim analysis 4, which occurs after 36 months’ follow-up, a total of 1000 participants have been randomized, and there is now sufficient follow-up. The intervention is eligible for both futility and success stopping since more than 100 patients have completed 108 weeks’ follow-up. The treatment effect is estimated to reduce UACR by 29.1% (95% CI, 22.7%-34.9%), and the probability of providing a clinically important 25% reduction is 0.899. The treatment effect on the chronic eGFR slope is 1.08 (95% CI, 0.23-1.93) mL/min/1.73 m^2^ per year. The posterior probability of a slope effect greater than 0 mL/min/1.73 m^2^ per year is 0.994 and meets the requirement to stop the intervention for success. The probability of a slope effect greater than 0.8 mL/min/1.73 m^2^ per year is 0.740. At this time, the domain is closed, and all patients discontinue the intervention and attend the end-of-treatment visit. Error bars indicate 95% CIs. In panels B and E, horizontal lines indicate the median change in UACR from baseline, and boxes indicate the IQR; the dots indicate outliers. CFB indicates change from baseline.

The first interim analysis assessing for futility is conducted once 100 patients in each treatment arm have completed 24 weeks of treatment using the change from baseline in albuminuria as an end point (Figure 2A-C). Once 100 participants in each treatment arm have completed 52 weeks of treatment, the change in eGFR from week 4 to week 52 (chronic eGFR change) will also be incorporated into the interim analysis (Figure 2 and Figure 3). The chronic eGFR change will be used as an end point since many of the participants will not have the washout period completed, which means that the eGFR change from baseline to washout cannot be used. Previous work has demonstrated that the change in eGFR during chronic treatment is the same as the change from baseline to the end of washout if the treatment effect is fully reversible.^27^ The use of the UACR for early futility assessment and eGFR for definitive efficacy assessment is an important feature of this trial design.

#### Sample Size

The study uses a default maximum sample size of 500 participants per treatment arm (1000 total for a single investigational arm and placebo). This allows for approximately 90% power to detect a clinically meaningful 1.3-mL/min/1.73 m^2^ per year (2.6 mL/min/1.73 m^2^ over a 2-year follow-up) improvement in the chronic eGFR slope between groups at week 108. A difference exceeding 2.0 mL/min/1.73 m^2^ over 2 years suggests a strong likelihood of reducing kidney failure risk and is considered clinically meaningful.^20^

#### Adaptive Design Elements

Interim analyses conducted approximately every 26 weeks will evaluate interventions for early stopping. Interventions may be stopped for futility if there is strong evidence that they do not provide a clinically meaningful benefit (at least 25% albuminuria reduction or a 0.8-mL/min/1.73 m^2^ per year eGFR slope improvement). Success is defined as a more than 0.985 posterior probability of superiority in eGFR vs placebo with at least 100 intervention patients (and 100 matched controls) completing a 108-week follow-up. This success threshold, determined using simulations, maintains the 1-sided type I error rate below .025, accounting for multiple analyses (Figure 2 and Figure 3). R, version 4.2.2 (R Project for Statistical Computing) was used for statistical analyses; stan version 2.26.1 (Rstan) was used for Markov chain Monte Carlo analysis.

## Results

### Patient Characteristics of GKPTN Participants at Baseline

Enrollment of patients in the GKPTN occurs at 119 clinical practice sites across 8 countries (US, Australia, Argentina, China, Italy, Canada, Spain, and Japan). At the time of data cutoff (March 2023), 4334 patients fulfilled all inclusion and exclusion criteria and were followed in the registry. Baseline demographics are summarized in Table 1. The mean (SD) age of participants at enrollment was 64.5 (16.2) years, 2542 (58.7%) were female, and 1791 (41.3%) were male. Most participants were White (2267 [52.3%]), followed by those who were Hispanic or Latino (866 [20.0%]); Black or African American (624 [14.4%]); Asian (533 [12.3%]); and American Indian or Alaska Native, Oceanian, or of other race or ethnicity (44 [1.0%]). Geographically, most patients were recruited from North America (2624 [60.5%]), followed by Europe (572 [13.2%]), South America (474 [10.9%]), Asia (464 [10.7%]), and Australia (200 [4.6%]).

**Table 1.** Baseline Characteristics of People Recruited to Date in the GKPTN

Characteristic	Total participants (N = 4334)^a^
Age, mean (SD), y	64.5 (16.2)
Sex, No. (%)	
Female	2542 (58.7)
Male	1791 (41.3)
Other	1 (0.02)
Race and ethnicity, No. (%)	
American Indian or Alaska Native, Oceanian,^b^ or other^c^	44 (1.0)
Asian	533 (12.3)
Black or African American	624 (14.4)
Hispanic or Latino	866 (20.0)
White	2267 (52.3)
Weight, mean (SD), kg	86.0 (22.6)
BMI, mean (SD)	30.5 (7.3)
Blood pressure, mean (SD), mm Hg	
Systolic	133.4 (17.3)
Diastolic	75.6 (10.6)
Type 2 diabetes diagnosis, No. (%)	2560 (59.1)
CKD etiology, No. (%)	
Diabetic kidney disease	1875 (43.3)
Glomerulonephritis	1026 (23.7)
Focal segmental glomerulosclerosis	182 (4.2)
IgA nephropathy	318 (7.3)
Membranous nephropathy	164 (3.8)
Minimal change disease	85 (2.0)
Hypertensive kidney disease	1330 (30.7)
Other	102 (2.4)
eGFR, mean (SD), mL/min/1.73 m^2^	52.9 (29.3)
≥60, No. (%)	1358 (31.3)
<60, No. (%)	2976 (68.7)
UACR, geometric mean (CV), mg/g	89 (20-420)
<30	1042 (24.0)
30-300	1172 (27.0)
>300 to ≤1000	595 (13.7)
>1000	424 (9.8)
Missing^d^	1101 (25.4)
Cardiovascular disease, No. (%)	1485 (34.3)
Concomitant medications, No. (%)	
ACEi or ARB use	3145 (72.6)
MRA use	86 (2.0)
SGLT2 inhibitor use	579 (13.4)

Abbreviations: ACEi, angiotensin converting enzyme inhibitor; ARB, angiotensin receptor blocker; BMI, body mass index (calculated as weight in kilograms divided by height in meters squared); CKD, chronic kidney disease; CV, coefficient of variation; eGFR, estimated glomerular filtration rate; GKPTN, Global Kidney Patient Trials Network; IgA, immunoglobulin A; MRA, mineralocorticoid receptor antagonist; SGLT2, sodium-glucose cotransporter-2; UACR, urinary albumin-to-creatinine ratio.

^a^ For data presented as No. (%) of people, percentages may not sum to 100 owing to rounding.

^b^ Includes Aboriginal people, Māori people, Native Hawaiian or Other Pacific Islander individuals, or Torres Strait Islander individuals.

^c^ Data did not specify categories for other race and ethnicity.

^d^ In 322 of these patients, UACR was recorded more than 1 year earlier from the date of baseline measurement (measurement performed within 1 year before to 6 months after the date of obtaining a signed informed consent form). In the case of more than 1 measurement in this period, the measurement performed closest to the date of obtaining a signed informed consent form was used.

With respect to kidney disease etiologies, diabetic kidney disease was the most frequently reported etiology among 1875 patients (43.3%). At baseline, the mean (SD) eGFR was 52.9 (29.3) mL/min/1.73 m^2^, and the median UACR was 89 mg/g (IQR, 20-420 mg/g). A total of 2976 participants (68.7%) had an eGFR less than 60 mL/min/1.73 m^2^. Regarding concomitant medications, angiotensin converting enzyme inhibitors or angiotensin receptor blockers were used in 3145 participants (72.6%) at baseline, while sodium-glucose cotransporter-2 inhibitors were used in 579 participants (13.4%).

### eGFR Decline During Follow-Up in the GKPTN Cohort

In the overall cohort during a median 0.89-year (IQR, 0.50-1.44–year) follow-up, the mean annual eGFR change was −1.67 (95% CI, −2.31 to −1.02) mL/min/1.73 m^2^ (Table 2). The rate of eGFR decline did not vary among age-specific or sex-specific subgroups (Table 2). In contrast, the mean eGFR decline was steeper among participants with a baseline eGFR of 60 mL/min/1.73 m^2^ or more (−2.29 [95% CI, −3.14 to −1.44]) compared with those with an eGFR of less than 60 mL/min/1.73 m^2^ (−1.16 [95% CI, −1.77 to −1.44]) and was progressively steeper in more severe albuminuria subgroups.

**Table 2.** eGFR Decline in the Overall GKPTN Cohort and Key Baseline Subgroups^a^

Subgroup	eGFR decline, mean (95% CI), mL/min/1.73 m^2^ per y	*P* value
Overall	−1.67 (−2.31 to −1.02)	NA
Age, y		
<65	−1.35 (−2.36 to −0.33)	.43
≥65	−1.87 (−2.70 to −1.04)	
Sex		
Female	−1.61 (−2.63 to −0.58)	.89
Male	−1.70 (−2.53 to −0.87)	
CKD etiology		
Diabetic kidney disease	−1.38 (−2.29 to −0.46)	.63
Glomerulonephritis	−1.89 (−4.05 to 0.28)	
Hypertensive kidney disease	−1.62 (−2.84 to −0.39)	
Other	−2.68 (−4.42 to −0.93)	
eGFR, mL/min/1.73 m^2^		
<60	−1.16 (−1.77 to −1.44)	.03
≥60	−2.29 (−3.14 to −1.44)	
UACR, mg/g		
<30	−0.34 (−1.46 to 0.79)	.001
30 to ≤300	−1.76 (−2.85 to −0.67)	
>300	−3.42 (−4.65 to −2.19)	
ACEi or ARB use		
No	−1.96 (−3.05 to −0.88)	.57
Yes	−1.60 (−2.25 to −0.95)	

Abbreviations: ACEi, angiotensin converting enzyme inhibitor; ARB, angiotensin receptor blocker; CKD, chronic kidney disease; eGFR, estimated glomerular filtration rate; GKPTN, Global Kidney Patient Trials Network; NA, not applicable; UACR, urinary albumin-to-creatinine ratio.

^a^ Includes all available data excluding those with withdrawn informed consent; therefore, the number of patients may differ among subgroups.

### Interim Analyses Within the CAPTIVATE Platform

The first interim, which is performed after at least 100 participants per treatment arm have completed the 6-month visit, occurs 18 months after the domain is open, when a total of 745 participants have been randomized and the intervention is assessed for futility based on the UACR end point. The treatment effect is estimated to reduce UACR by 31.2% (95% CI, 21.9%-39.3%), and the probability of providing a clinically important 25% reduction is 0.909; therefore, futility is not declared for the intervention, and the trial continues to recruit. Although there is some preliminary data suggesting that the treatment also reduces the eGFR slope (difference in eGFR slope, 1.11 [95% CI, −1.73 to 3.95] mL/min/1.73 m^2^), there is insufficient follow-up to assess either success or futility on this end point. The probability of a slope effect greater than 0 mL/min/1.73 m^2^ per year is 0.779; the probability of a slope effect greater than 0.8 mL/min/1.73 m^2^ per year is 0.586 (Figure 2A-C).

The next interim analysis is performed at 24 months. Enrollment is complete, and the domain has reached the maximum sample size of 1000. The treatment continues to show a strong benefit on UACR and does not meet the futility criteria. The treatment effect is estimated to reduce UACR by 28.4% (95% CI, 21.2%-35.0%), and the probability of providing a clinically important 25% reduction is 0.830. The intervention is eligible for futility stopping on the eGFR end point, but the probability of providing a clinically important 0.8 mL/min/1.73 m^2^ per year difference compared with placebo in the eGFR slope is 0.778, and so the intervention is not stopped for futility, and the trial continues to recruit. The difference in the eGFR slope is 1.38 [95% CI, −0.11 to 2.88] mL/min/1.73 m^2^. The probability of a slope effect greater than 0 mL/min/1.73 m^2^ per year is 0.965 (Figure 2D-F).

At the third interim analysis at 30 months, a total of 1000 participants have been randomized, and the treatment continues to show a strong benefit on UACR (UACR reduction, 29.1% [95% CI, 22.7%-34.9%]; probability of providing a clinically important 25% reduction, 0.899) and the eGFR slope (1.32 [95% CI, 0.24-2.39] mL/min/1.73 m^2^ per year). While the posterior probability of superiority on the eGFR is above the 0.985 threshold (probability of slope effect >0 mL/min/1.73 m^2^ per year, 0.992), the number of participants assigned to the intervention with a complete 108-week follow-up is less than 100, and so the intervention is not eligible to stop for success, and the trial continues. The probability of a slope effect greater than 0.8 mL/min/1.73 m^2^ per year is 0.826 (Figure 3A-C).

At the fourth interim analysis, performed at 36 months with a total of 1000 participants who have been randomized, there is now sufficient follow-up, and the intervention is eligible for both futility and success stopping since more than 100 patients have completed 108 weeks of follow-up. The treatment effect is estimated to reduce UACR by 29.1% (95% CI, 22.7%-34.9%), and the probability of providing a clinically important 25% reduction is 0.899. The treatment effect on the chronic eGFR slope is 1.08 (95% CI, 0.23-1.93) mL/min/1.73 m^2^ per year. The posterior probability of a slope effect greater than 0 mL/min/1.73 m^2^ per year is 0.994 and meets the requirement to stop the intervention for success. The probability of a slope effect greater than 0.8 mL/min/1.73 m^2^ per year is 0.740. At this time, the domain is closed, and all patients discontinue the intervention and attend the end-of-treatment visit (Figure 3D-F).

## Discussion

We successfully established a large global registry of patients with CKD and an adaptive platform clinical trial to assess multiple interventions to attempt to slow CKD progression. Combining a registry with a platform trial facilitates patient access and enables timely efficacy and safety assessment of novel therapeutic agents. The master protocol will begin with a mineralocorticoid receptor antagonist. Addition of future domains will allow head-to-head comparisons of novel interventions and identify potential complementary or synergistic effects, guiding tailored strategies for individual or combination therapies. Factorial randomization will help determine optimal combinations for specific patient subgroups.

The GKPTN registry provides access to a large, diverse patient population crucial for platform trials. Over half of the participants were female, and almost half belonged to minoritized racial and ethnic groups, which enhanced generalizability. Data from diverse regions (US, Australia, Argentina, China, Italy, Canada, Spain, and Japan) reflect standard nephrology practices, highlighting the lower rates of kidney-protective therapies. Ongoing data collection will also track prescription pattern changes. eGFR decline rates within the GKPTN are comparable with other cohorts (The Study of Heart and Kidney Protection With Empagliflozin [EMPA-Kidney] trial,^28^ the Chronic Renal Insufficiency Cohort [CRIC] study,^29^ and The German Chronic Kidney Diseases [GCKD] study),^30,31^ which further validates the registry’s representativeness.

CAPTIVATE’s primary end point is eGFR change, a validated surrogate end point for kidney failure to establish drug efficacy. A meta-analysis involving 66 randomized clinical trials and more than 160 000 participants demonstrated that therapies slowing eGFR decline by 0.75 mL/min/1.73 m^2^ per year have a more than 99% likelihood of reducing kidney failure risk.^20^ Some interventions produce early, reversible reductions in eGFR that differ from their long-term treatment effects on the progressive loss of eGFR over time. These early acute reductions are reversed after drug discontinuation and often reflect a glomerular hemodynamic effect but can also complicate the long-term assessment of drug efficacy. To address this, CAPTIVATE’s primary end point is defined as the change in eGFR from baseline to the end of a 4-week washout period. This is similar to other ongoing phase 3 registration trials in nephrology (Atrasentan in Patients With IgA [Immunoglobulin A] Nephropathy [ALIGN]^32^ and A Phase III Study to Investigate the Efficacy and Safety of Baxdrostat in Combination With Dapagliflozin on CKD Progression in Participants With CKD and High Blood Pressure [ARCTIC]).^33^ This approach, validated by analyses of past trials, allows for accurate assessment of treatment effects on the eGFR slope.^27^

Emerging data suggest that 6-month albuminuria change is a reasonable surrogate end point for drug efficacy, with meta-analyses of randomized clinical trials demonstrating a strong link to clinical outcomes.^20,34^ The conservative futility analyses rules, including the change in albuminuria used in the CAPTIVATE trial, enable us to identify nonefficacious interventions early while also protecting against declaring a lack of benefit for potentially beneficial therapies.

The design of the CAPTIVATE trial efficiently identifies new treatments for CKD. Its platform structure both obviates the need for separate trials for each intervention and leverages economies of scale when evaluating multiple interventions simultaneously.^35^ The factorial randomization scheme allows participants to be randomized to multiple interventions, mirroring clinical practice and enabling the study of interactions. The adaptive elements efficiently stop ineffective drugs while allowing success to be declared early for beneficial interventions. According to our findings, stopping futile interventions prevents patient exposure to ineffective drugs and reduces the average time to decision by 27 to 36 months and the average sample size allocated to that intervention by approximately 20% to 50% depending on the accrual rate. Together, these design features create a perpetual engine of discovery for CKD interventions.

### Limitations

A limitation of the GKPTN is that the patient selection and data collection depend on site investigators, which could lead to missing data and waning interest over time. Selection bias is possible, as only patients interested in participating in clinical trials are enrolled in the GKPTN, which may not be reflective of all patients with CKD. The pragmatic nature of the GKPTN and the use of local laboratory testing can lead to discrepancies in consistency and standardization but on the other hand reflect current clinical practice. The operational implementation and statistical design complexities of the CAPTIVATE trial pose challenges. Our inclusion of all patients with CKD is likely to introduce heterogeneity in the patient cohort included. However, despite these limitations, the CAPTIVATE platform holds significant promise for accelerating treatment options for people with kidney disease.

## Conclusions

To our knowledge, CAPTIVATE is the first adaptive platform randomized clinical trial testing combination therapies in people with kidney disease and represents a paradigm shift in clinical trials in nephrology. The GKPTN and CAPTIVATE exemplify a shift in nephrology toward increased therapeutic advances and create opportunities for personalized medicine and combination therapy. With multiple new therapies becoming available for patients with CKD, there is an opportunity to personalize treatments in individual patients with CKD, but there are minimal head-to-head comparisons of available therapeutic agents or of available data on the best combinations. To efficiently improve outcomes of people with CKD with these newly identified therapies, it is essential to evaluate the benefits of these new therapies over and above standard of care to determine if there are any synergistic effects and to develop guidance for directing specific types of therapies to specific patients in a timely fashion. This approach has the potential to revolutionize clinical trial conduct in nephrology, accelerating therapeutic development for patients with CKD.

## Notes

Supplement 1. **eTable 1.** Inclusion and Exclusion Criteria for the GKPTN Cohort Study **eTable 2.** Primary and Secondary Outcomes for the CAPTIVATE Platform

Supplement 2.
Trial Protocol

Supplement 3.
Nonauthor Collaborators

Supplement 4.
Data Sharing Statement
