# Supplementary material for: The Global Kidney Patient Trials Network and the CAPTIVATE Platform Clinical Trial Design: A Trial Protocol
Source: JAMA Netw Open. 2024 Dec 11;7(12):e2449998. doi: 10.1001/jamanetworkopen.2024.49998 (PMC11635535; doi:10.1001/jamanetworkopen.2024.49998)
Supplement: Supplement 1. — eTable 1. Inclusion and Exclusion Criteria for the GKPTN Cohort Study eTable 2. Primary and Secondary Outcomes for the CAPTIVATE Platform [file jamanetwopen-e2449998-s001.pdf]

Supplemental Online Content

Kotwal SS, Perkovic V, Jardine MJ, et al. The Global Kidney Patient Trials Network and the CAPTIVATE platform clinical trial design: a trial protocol. *JAMA Netw Open*. 2024;7(12):e2449998. doi:10.1001/jamanetworkopen.2024.49998

eTable 1. Inclusion and Exclusion Criteria for the GKPTN Cohort Study

eTable 2. Primary and Secondary Outcomes for the CAPTIVATE Platform

This supplemental material has been provided by the authors to give readers additional information about their work.

© 2024 Kotwal SS et al. *JAMA Network Open*.

eTable 1. Inclusion and Exclusion Criteria for the GKPTN Cohort Study

Inclusion and Exclusion Criteria for GKPTN
**Inclusion criteria**
1. Documented diagnosis of primary kidney disease
a. Diabetic Kidney Disease
b. Hypertensive Kidney Disease
c. Focal Segmental Glomerulosclerosis (FSGS)
d. Membranous Nephropathy
e. Minimal Change Disease
f. IgA Nephropathy
g. Glomerulonephritis not otherwise specified.
h. Other
2. Regular visits (6 - 12 monthly) with a physician at a participating site that includes the monitoring of protein in urine and kidney function (refer to section 3.3)
3. eGFR > 15 ml/min/1.73m2
4. ≥ 2 years of age
5. Willing and able to sign informed consent.
6. Willing to be approached about participation in interventional research studies.
**Exclusion criteria**
1. Life-expectancy of less than 6 months
2. Currently receiving kidney replacement therapy (maintenance dialysis or kidney transplantation) or planned to start in the next 6 months
3. Currently participating in a clinical trial involving investigational medicinal product (exception is participation in studies implemented through the GKPTN)

© 2024 Kotwal SS et al. *JAMA Network Open*.

eTable 2. Primary and Secondary Outcomes for the CAPTIVATE Platform

Variable	Definition
Primary outcome	Chronic eGFR slope calculated using all available eGFR values from week 4 to week 104
Secondary outcomes that will be evaluated are:	1. Change in albuminuria as measured by uACR (or uPCR if uACR unavailable) between randomisation and 24 weeks, measured as a continuous variable.<br>2. Change in eGFR from randomisation to end of washout<br>3. Composite outcome of proportion of participants experiencing a 40% eGFR decline between randomisation and 108 weeks, and proportion of participants developing kidney failure (defined as eGFR <15 mL/min/1.73m2 or chronic kidney replacement therapy start) at 108 weeks.<br>4. Time to a composite outcome of ≥40% eGFR decline from randomisation or kidney failure<br>5. All-cause mortality at 108 weeks<br>6. Proportion of participants experiencing one or more cardiovascular events (cardiovascular death, hospitalised heart failure, myocardial infarction, stroke) between randomisation and 108 weeks<br>7. Time to first occurrence of a cardiovascular event<br>8. Safety and tolerability of treatment<br>9. Change in quality of life measured using the Quality-of-Life Impact Survey for Kidney Disease (QDIS-CKD) at 6-monthly intervals from randomisation to week 108.
Exploratory outcomes	Health care resource utilization and costs<br>Win ratios which may include, but are not limited to:<br>• All-cause mortality<br>• Proportion of participants developing kidney failure<br>• Proportion of participants experiencing a 40% eGFR decline<br>• Rate of eGFR decline

© 2024 Kotwal SS et al. *JAMA Network Open*.
