# Supplementary material for: The Global Kidney Patient Trials Network and the CAPTIVATE Platform Clinical Trial Design: A Trial Protocol
Source: JAMA Netw Open. 2024 Dec 11;7(12):e2449998. doi: 10.1001/jamanetworkopen.2024.49998 (PMC11635535; doi:10.1001/jamanetworkopen.2024.49998)
Supplement: Supplement 2. — Trial Protocol [file jamanetwopen-e2449998-s002.pdf]

The Chronic kidney disease Adaptive Platform Trial Investigating Various Agents for Therapeutic Effect (CAPTIVATE)

Core Protocol
V2.0, 16 November 2023

Protocol Number: P01351

Name: Sradha Kotwal
Role: Platform Chief Investigator
Signature:

Name: Hiddo Lambers-Heerspink
Role: Platform Chief Investigator
Signature:

PRIVILEGED AND CONFIDENTIAL
Not for distribution beyond intended function

1 TABLE OF CONTENTS

1 TABLE OF CONTENTS 2
2 ABBREVIATIONS AND GLOSSARY 5
2.1 Abbreviations 5
2.2 Glossary 6
3 INTRODUCTION 8
3.1 Trial Synopsis 8
3.2 Key Administrative Information 10
3.2.1 Sponsor 10
3.2.2 Funding 10
3.2.3 Registration 10
3.2.4 Protocol Development 10
3.2.5 Chief Investigators 10
3.3 Protocol Structure 11
3.3.1 Core Protocol 11
3.3.2 Domain-Specific Appendix (DSA) 11
3.3.3 Country-Specific Appendix (CSA) 12
3.3.4 Statistical Analysis Appendix 12
3.3.5 Standard of Care Appendix 12
4 TRIAL GOVERNANCE 13
4.1 Oversight 13
4.1.1 Platform Oversight Committee 13
4.1.2 DSA Steering Committee 13
4.1.3 Statistics & Methodology Committee 13
4.1.4 Data Safety Monitoring Board 14
4.1.5 Consumer Engagement 14
4.1.6 Other Committees 14
4.1.7 Trial Organigram 15
4.2 Trial Coordination 15
5 BACKGROUND & RATIONALE 16
5.1 Introduction and Setting 16
5.1.1 Burden of Chronic Kidney Disease 16
5.1.2 Standard of Care for Patients with CKD 17
5.1.3 Study Rationale 18
5.1.4 Valid Surrogate Kidney Endpoints 18
5.2 Adaptive Platform Trials 19
6 OBJECTIVES 21
6.1 Primary Objective 21
6.2 Secondary Objectives 21
6.3 Tertiary Objectives 21
6.4 Exploratory Objectives 21
6.5 DSA-Specific Objectives 21
7 TRIAL DESIGN 22
7.1 Overview 22
7.2 Trial Setting 22
7.3 Platform Eligibility Criteria 22

7.3.1 Inclusion Criteria 22
7.3.2 Exclusion Criteria 22
7.3.3 Domain-Specific Criteria 23
7.4 Interventions 23
7.5 Outcomes 24
7.5.1 Primary Outcome 24
7.5.2 Secondary Outcomes 24
7.5.3 Tertiary Outcomes 25
7.5.4 Exploratory Outcomes 25
8 TRIAL CONDUCT 26
8.1 Consent 26
8.1.1 Trial Consent 26
8.1.2 Withdrawal of Consent 26
8.2 Participant Schedule 27
8.2.1 Screening (Day -31 to Day -1) 29
8.2.2 Randomisation (Day 0) 29
8.2.3 Active Follow-Up 30
8.2.4 End of Treatment 30
8.2.5 Final Visit 30
8.2.6 Early Treatment Discontinuation Visit (4 weeks after early discontinuation of treatment)...30
8.2.7 Passive Follow-Up 31
8.2.8 Site Transfer 31
8.2.9 Loss to Follow-Up 31
8.2.10Participant Discontinuation from Trial 31
8.3 Data Linkage 32
8.4 Management of Pandemics & Exceptional Circumstances 32
8.5 Blinding & Unblinding 32
8.6 Treatment of CKD 33
8.6.1 Standard of Care 33
8.6.2 Protocol Study Interventions 33
8.7 DSA Closure 33
8.8 End of Trial 34
8.9 Post-Trial Care 34
9 DATA MANAGEMENT 35
9.1 Data Collection & Handling 35
9.1.1 Archiving 35
9.2 Data Sharing 35
9.2.1 Data Ownership 35
9.2.2 Data Dissemination 35
9.2.3 Data Release Requests 36
10 QUALITY MANAGEMENT 37
10.1 Site Management 37
10.1.1Selection & Feasibility 37
10.1.2Training 37
10.1.3Site Documents 37
10.2 Quality Assurance 37
10.2.1Central Monitoring 38
10.2.2On Site Monitoring 38

10.2.3 Audit & Inspection 38
10.3 Protocol Deviations 38
11 SAFETY MANAGEMENT 39
11.1 Definitions 39
11.1.1 Adverse Event 39
11.1.2 Adverse Reaction 39
11.1.3 Serious Adverse Event 39
11.1.4 Serious Adverse Reaction 40
11.1.5 Suspected Unexpected Serious Adverse Reaction 40
11.1.6 Adverse Events of Special interest 40
11.1.7 Significant Safety Issues & Urgent Safety Measures 40
11.2 Assessments 41
11.2.1 Causality 41
11.2.2 Expectedness 41
11.3 Pregnancy 41
11.4 Reporting 42
11.4.1 Event Reports 42
11.4.2 Expedited Reporting 42
11.4.3 Non-Expedited Reporting 42
11.4.4 Reporting of Adverse Events of Special Interest 43
11.4.5 Exemptions to Reporting 43
11.4.6 Safety Reports 43
12 STATISTICAL CONSIDERATIONS 44
12.1 Platform Trial Overview 44
12.2 Statistical Analysis Overview 44
12.3 Sample Size 45
12.4 Randomisation Principles 45
12.5 Analyses 45
12.5.1 Primary Analysis Population 46
12.5.2 Primary Analysis 46
12.5.3 uACR Analysis 46
12.5.4 Interim Analysis 46
12.5.5 Changes in the Standard of Care 47
13 ETHICAL CONSIDERATIONS 48
13.1 Ethics & Regulatory Compliance 48
13.2 Confidentiality 48
13.3 Insurance 48
13.4 Declaration of Interests 48
14 REFERENCES 50
15 PROTOCOL AMENDMENTS 53
16 APPENDIX 54
16.1 Outcome Definitions for Cardiovascular Events 54
16.1.1 Cardiovascular (CV) death (1) 54
16.1.2 Hospitalised heart failure (1, 2) 54
16.1.3 Myocardial infarction (MI) (1, 3) 55
16.1.4 Stroke (1, 4) 56
16.2 Quality of Life Impact Survey for Kidney Disease (QDIS-CKD) 57

2 ABBREVIATIONS AND GLOSSARY

2.1 Abbreviations

Abbreviation	Definition
ACEi	Angiotensin-Converting Enzyme inhibitor
AE	Adverse Event
AESI	Adverse Event of Special Interest
AKI	Acute Kidney Injury
AR	Adverse Reaction
ARB	Angiotensin Receptor Blocker
BRC	Biomarker & Research Committee
CEC	Consumer Engagement Committee
CKD	Chronic Kidney Disease
CKD-EPI	Chronic Kidney Disease Epidemiology Collaboration
CRFs	Case Report Forms
CSA	Country-Specific Appendix
CV	Cardiovascular
DSA	Domain-Specific Appendix
DSC	Domain-Specific Appendix Steering Committee
DSMB	Data Safety Monitoring Board
DSUR	Development Safety Update Report
ECG	Electrocardiogram
eCRF	Electronic Case Report Form
EDC	Electronic Data Capture
eGFR	Estimated Glomerular Filtration Rate
EMA	European Medicines Agency
EOT	End of Treatment
ETDV	Early Treatment Discontinuation Visit
FDA	Food and Drug Administration
GCC	Global Coordinating Centre
GCP	Good Clinical Practice
HREC	Human Research Ethics Committee
IRB	Institutional Review Board
ISF	Investigator Site File
KDIGO	Kidney Disease Improving Global Outcomes
KRT	Kidney Replacement Therapy
MACE	Major Adverse Cardiovascular Events
MI	Myocardial Infarction
MRA	Mineralocorticoid Receptor Antagonist
NHMRC	National Health and Medical Research Council
NICE	National Institute for Health and Care Excellence
PISCF	Participant Information Sheet and Consent Form
POC	Platform Oversight Committee
QDIS-CKD	Quality of Life Impact Survey for Kidney Disease
QMMP	Quality Management & Monitoring Plan

Abbreviation	Definition
RAR	Response Adaptive Randomisation
RAS	Renin-Angiotensin System
RCC	Regional Coordinating Centre
RCT	Randomised Controlled Trial
RSI	Reference Safety Information
SAE	Serious Adverse Event
SAR	Serious Adverse Reaction
SGLT2i	Sodium-Glucose Cotransporter-2 Inhibitor
SMC	Statistics & Methodology Committee
SSI	Significant Safety Issue
SUSAR	Suspected Unexpected Serious Adverse Reaction
TGI	The George Institute for Global Health
TTSC	Therapeutics & Trial Selection Committee
uACR	Urine Albumin-Creatine Ratio
uPCR	Urine Protein-Creatinine Ratio
USM	Urgent Safety Measure

2.2 Glossary

Adaptive Platform Trial: A clinical trial in which multiple interventions are evaluated in a perpetual manner using a single overarching infrastructure, and with flexible pre-defined design features that allow inventions to be dropped for futility, declared superior, or added during the course of the trial.

Core Protocol: A module of the CAPTIVATE protocol that details the information that applies to all interventions and countries/regions that are involved in the trial. This module defines the overall infrastructure of the platform trial and should be implemented in conjunction with the relevant Domain-Specific Appendices and Country-Specific Appendix.

Country-Specific Appendix: A module of the CAPTIVATE protocol that details the specific aspects of trial design and implementation of the platform within a specific country or region. This module should be implemented in conjunction with the Core Protocol and relevant Domain-Specific Appendices.

Intervention: A treatment option that is being subjected to experimental manipulation within the design of the CAPTIVATE protocol.

Domain-Specific Appendix: A module of the CAPTIVATE protocol that details the specific aspects of trial design and implementation for the interventions that are nested within a domain of the CAPTIVATE platform infrastructure. This module should be implimented in conjunction with the Core Protocol and the relevant Country-Specific Appendix.

Response Adaptive Randomisation: A randomisation procedure that uses past intervention assignments and participants responses to alter the probability of allocation to different intervention arms. Typically favours better performing intervention arms at the time the calculation is made.

Standard of Care: The treatment process that a physician should follow for a certain type of patient, illness or clinical circumstance, as documented in relevant local guidelines for patient, illness or clinical circumstance management.

3 INTRODUCTION

3.1 Trial Synopsis

**Sponsor**	The George Institute for Global Health
**Project Title**	The Chronic kidney disease Adaptive Platform Trial Investigating Various Agents for Therapeutic Effect (CAPTIVATE)
**Chief Investigators**	Sradha Kotwal; Hiddo Lambers-Heerspink
**Sponsor**	The George Institute for Global Health
**Clinical Phase**	Phase III
**Design**	Adaptive, platform, randomised controlled trial
**Primary Objective**	To determine investigational agents or combinations of agents that reduce the rate of eGFR decline (slow progression of CKD), compared to placebo, in patients with chronic kidney disease receiving standard of care therapy.
**Platform Primary Outcome**	eGFR slope calculated using eGFR values from randomisation to week 108
**Platform Secondary Outcomes**	1. Change in albuminuria as measured by uACR (or uPCR if uACR unavailable) between randomisation and 24 weeks, measured as a continuous variable<br>2. Composite outcome of proportion of participants experiencing a 40% eGFR decline between randomisation and 108 weeks, and proportion of participants developing kidney failure (defined as eGFR <15 mL/min/1.73m2 or chronic kidney replacement therapy start) at 108 weeks<br>3. Time to a composite outcome of ≥40% eGFR decline from randomisation or kidney failure<br>4. All-cause mortality at 108 weeks<br>5. Proportion of participants experiencing one or more cardiovascular events (cardiovascular death, hospitalised heart failure, myocardial infarction, stroke) between randomisation and 108 weeks<br>6. Time to first occurrence of a cardiovascular event<br>7. Safety and tolerability of treatment<br>8. Change in quality of life measured using the Quality of Life Impact Survey for Kidney Disease (QDIS-CKD) at 6-monthly intervals from randomisation to week 108
**Platform Tertiary Outcomes**	1. All-cause mortality assessed at 5 years and at subsequent 5-year intervals<br>2. Proportion of participants who have developed kidney failure assessed at 5 years and at subsequent 5-year intervals, as defined by requirement for chronic kidney replacement therapy or kidney transplant surgery
**Platform Exploratory Outcomes**	1. Health care resource utilisation and costs.<br>2. Win ratio approach to evaluating composite outcomes.
**Platform Inclusion Criteria**	Potential participants must satisfy all of the following:<br>1. Age ≥ 18 years<br>2. Known chronic kidney disease from any cause (eGFR ≥25 mL/min/1.73m2)

	3. Currently receiving standard of care treatment according to treating physician<br>4. Eligible for randomisation in at least one recruiting domain-specific appendix<br>5. Participant and treating physician are willing and able to perform trial procedures
**Platform Exclusion Criteria**	Potential participants must have none of the following:<br>1. Currently receiving maintenance dialysis<br>2. Planned to commence kidney replacement therapy or kidney transplant surgery in next 6 months<br>3. Life expectancy less than 6 months
**Sample Size**	Each intervention will have a maximum sample size specified within the associated domain-specific appendix (DSA). The default sample size is 500 participants per intervention, which provides approximately 90% power to detect a 1.3 mL/min/1.73m2/year improvement in eGFR slope. The total sample size of a domain is obtained by summing up the individual interventions; a domain with a single intervention and a control will have a maximum sample size of approximately 1,000 participants. The final sample size will depend on the simulations conducted prior to the DSA implantation.
**Platform Duration**	Ongoing and in perpetuity.<br>Participants in each DSA will be actively followed up for 108 weeks following randomisation, and passively followed up until death. Should specific interventions require additional follow-up, this will be specified within the relevant DSA.
**Intervention**	Unless specified within the relevant DSA, participants will be randomised to receive one intervention within each DSA.
**Consumer Engagement**	Consumer feedback has been incorporated in the trial design and consumers will continue to actively engage in the design and implementation of the trial. At a minimum, ongoing input into trial activities and participant-facing materials will be facilitated via the Kidney Consumer Panel at The George Institute for Global Health.

3.2 Key Administrative Information

3.2.1 Sponsor

The George Institute for Global Health (TGI) is the sponsor of CAPTIVATE.

**Name:**	**The George Institute for Global Health**	
**Address:**	To 18 November 2023:<br>Level 5, 1 King Street<br>Newtown NSW 2050<br>Australia	From 19 November 2023:<br>Level 18, International Tower 3,<br>300 Barangaroo Ave<br>Sydney NSW 2000<br>Australia
**Contact:**	+61 2 8052 4300 (Reception)	
**Website:**	www.georgeinstitute.org.au	

3.2.2 Funding

The trial will be funded through multiple sources including peer-reviewed and pharmaceutical sources. Principle funding is via seed funding through The George Institute for Global Health and the Australian Government National Health and Medical Research Council (NHMRC) Clinical Trials and Cohort Studies grant (Grant Identification Number 2024079). Additional peer-reviewed funding will be sought from other participating countries and through industry collaborators where appropriate. All funding applications should be approved by the Global Coordinating Centre (GCC) prior to their submission.

3.2.3 Registration

This trial is registered on the ClinicalTrials.gov clinical trials register (ClinicalTrials.gov Identifier: NCT06058585).

3.2.4 Protocol Development

The protocol has been developed by TGI and the Platform Oversight Committee (POC). This core protocol, all appendices and future versions will be approved by the Sponsor, and POC prior to implementation.

3.2.5 Chief Investigators

Platform Chief Investigators

Dr Sradha Kotwal, The George Institute for Global Health
Prof Hiddo Lambers-Heerspink, The George Institute for Global Health

3.3 Protocol Structure

The CAPTIVATE platform trial uses a 'modular' protocol design that allows the trial to be highly adaptive and to evolve over time using pre-specified rules. Treatments are organized into domains, which consist of multiple mutually exclusive treatment options, referred to as interventions. Adaptations include the introduction or removal of interventions and domains, addition of new countries and the potential use of response adaptive randomisation.

The CAPTIVATE protocol consists of a Core Protocol, multiple Domain-Specific Appendices (DSAs), multiple Country-Specific Appendices (CSAs), a Statistical Analysis Appendix and a Standard of Care Appendix (Figure 1). This design allows interventions and countries to be introduced or removed without altering the Core Protocol. Trial features, procedures or ethical considerations that are specific to a particular domain or country/region are outlined separately in the respective appendices.

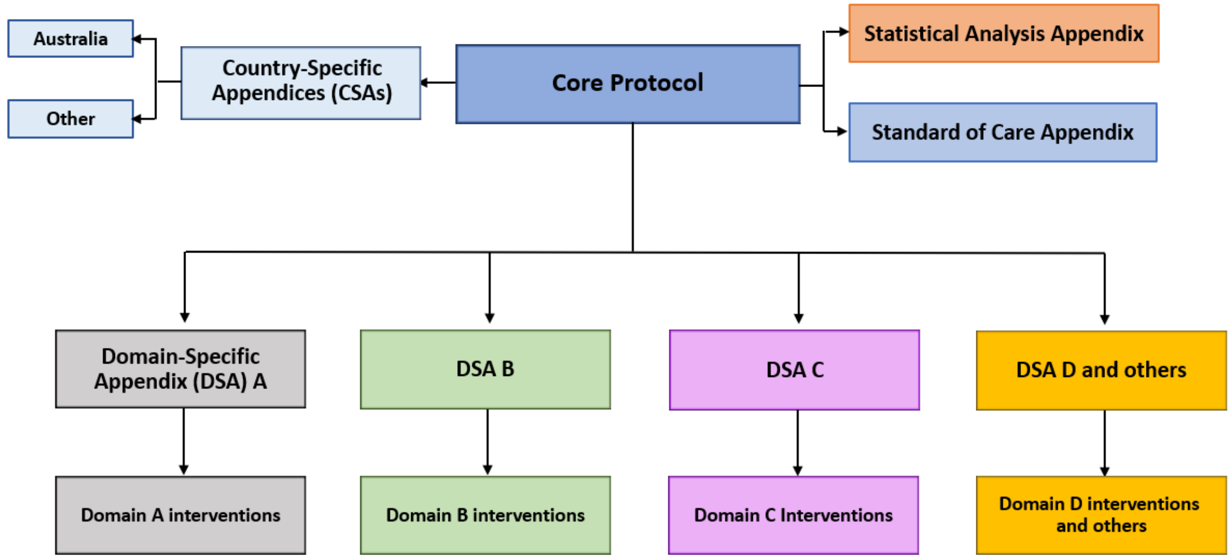

Figure 1: Structure of CAPTIVATE

3.3.1 Core Protocol

The Core Protocol contains information that applies to all domains and countries involved in the platform trial, including the trial background, design, procedures, outcomes (efficacy and safety), principles of statistical analyses, trial governance and ethical considerations. The Core Protocol may be amended but it is anticipated that such amendments will be infrequent. It does not include information about specific interventions that will be investigated in the CAPTIVATE trial.

3.3.2 Domain-Specific Appendix (DSA)

A DSA will be constructed for the interventions nested within a domain that are to be integrated into the CAPTIVATE trial. All information provided in the DSA is in addition to, and never instead of, that provided in the Core Protocol. The DSA will contain all information that is specific to the interventions, including any domain-specific eligibility criteria or procedures. Appendices to the DSA may be constructed to provide additional information on a specific aspect of the DSA, such as clinical guidance for administering the interventions. DSAs are anticipated to change over time. DSAs may be amended to add or remove specific interventions. In addition, entire DSAs may also be added or removed. In all instances, additional ethical approval will be sought.

3.3.3 Country-Specific Appendix (CSA)

CAPTIVATE is a global trial with sites in multiple countries. Where required by variation in local practice and regulations, a CSA may be created for individual countries or regions. CSAs may be added or amended over time. Each CSA contains information on trial conduct that is specific to the country or region, as a supplement to details provided in the Core Protocol. Information provided in the CSAs includes, but is not limited to:

- Availability of interventions in the country or region
- Trial governance and administration in the country or region
- Ethical issues that are specific to the country or region

3.3.4 Statistical Analysis Appendix

The Statistical Analysis Appendix contains a detailed description of how the statistical analysis will be conducted. The appendix is anticipated to change over time as trial adaptations occur and new interventions are added. The Statistical Analysis Appendix for CAPTIVATE is the Core Statistical Design and Simulation Report.

3.3.5 Standard of Care Appendix

The Standard of Care Appendix contains information about standard of care treatment for patients with chronic kidney disease (CKD) based clinical guideline recommendations. Should there be variations in local guideline recommendations in individual countries or regions, these will be described in the appendix. The Standard of Care Appendix will be amended as guidelines are updated.

4 TRIAL GOVERNANCE

4.1 Oversight

The trial governance structure is designed to provide appropriate management of all aspects of the trial. It includes representation from regions that are participating in the trial, skills and expertise related to trial conduct and statistical analysis, and content knowledge about CKD and trial interventions.

4.1.1 Platform Oversight Committee

The Platform Oversight Committee (POC) will take overall responsibility for the design and conduct of the trial. The POC will comprise the Platform Chief Investigators, a trial statistician, selected National Leads, invited experts and an operational staff member from the GCC. Invited experts will include consumer representatives. Members will have relevant knowledge and experience in trial design, domain-specific expertise, region-specific expertise and/or consumer specific expertise. The POC will be responsible for:

- Development and updates to the Core Protocol
- Consideration and approval of new countries/regions
- Consideration and approval of new interventions to add to CAPTIVATE
- Liaise with DSMB
- Contribute to interpretation and reporting of results from DSAs, in consultation with DSA Steering Committee
- Oversee and approve publications from CAPTIVATE
- Approval of data release requests
- Obtain funding for CAPTIVATE
- Determining the strategic direction of CAPTIVATE

The POC's roles and responsibilities will be described in detail in a POC charter.

4.1.2 DSA Steering Committee

Each DSA will be administered by a DSA Steering Committee (DSC). A single DSC may have oversight of more than one DSA. The DSC provides supervision and is responsible for the design and conduct of the DSA. Each country or region involved in a DSA will have a National Lead or a representative on the relevant DSC. The DSC is accountable to the POC.

Responsibilities of the DSC include:

- Development and updates to the DSA
- Interpret and report results from DSA, in consultation with POC.
- Obtain funding to support the DSA and where possible, contribute to the conduct of CAPTIVATE
- DSA-specific updates to the CSA(s) for the country or region
- Consider the feasibility and suitability of DSA for the country or region
- Identification and management of sites for the DSA in the country or region
- Convene country or region-specific Consumer Engagement Committees as required

The DSC's roles and responsibilities will be described in detail in a DSC charter.

4.1.3 Statistics & Methodology Committee

The Statistics & Methodology Committee (SMC) will comprise statisticians and triallists with experience in relevant statistical concepts and adaptive trials methodology. The SMC is responsible for:

- Overall statistical approach for CAPTIVATE

- Provision of advice on statistics and methodology
- Provision of advice on adaptive design features

Roles and responsibilities of the SMC will be described in detail in a SMC Charter.

4.1.4 Data Safety Monitoring Board

A single independent DSMB for the trial will be convened by the Sponsor and operate in accordance with the CAPTIVATE DSMB Charter. This charter will describe the groups' structure, roles and responsibilities, including their remit to protect the safety of trial participants and the scientific integrity of the trial by monitoring accumulating safety and operational data. The DSMB will make appropriate recommendations to the POC regarding trial continuation and modifications to trial design and procedures while maintaining confidentiality of the accumulating data. The POC will retain sole decision-making responsibility for modifications to or early stopping of the trial.

4.1.5 Consumer Engagement

Consumer feedback has been incorporated in the trial design and consumers will continue to actively engage in the design and implementation of the trial. At a minimum, ongoing input into trial activities and all participant-facing materials will be facilitated by the Kidney Consumer Panel at The George Institute for Global Health. Other avenues for consumer feedback may be implemented, particularly in countries outside Australia.

Consumer Engagement Committees (CECs) will be convened by DSCs as required in individual regions to provide region-specific input into trial activities and materials.

4.1.6 Other Committees

A number of other committees may be considered and convened in the future should the need arise. In the absence of these committees, their responsibilities will be undertaken by the POC or the GCC. Should any of these committees be convened, their roles and responsibilities will be described in detail in the relevant committee charter. These include but are not limited to:

- **Therapeutics & Trial Selection Committee (TTSC)** that can provide advice to the POC of potential interventions that can be incorporated into CAPTIVATE based on pre-specified intervention-assessment criteria. The TTSC can provide advice on the timing and prioritisation for the addition of new interventions. Members of the TTSC will have expertise across a range of interventions. In the absence of a TTSC, the POC will undertake this role.
- **Quality & Safety Committee (QSC)** that provides oversight of pharmacovigilance and participant safety for CAPTIVATE. Members of the QSC will include trialists with significant experience in quality and safety management. The GCC will manage pharmacovigilance and participant safety in the absence of a QSC, including the recruitment and management of medical monitors.
- **Biomarker & Research Committee (BRC)** that can provide advice to the POC of potential biomarker research that can be incorporated into CAPTIVATE. The BRC can also provide advice on the use of data from CAPTIVATE for research purposes. In the absence of a TTSC, the POC will undertake this role.

4.1.7 Trial Organigram

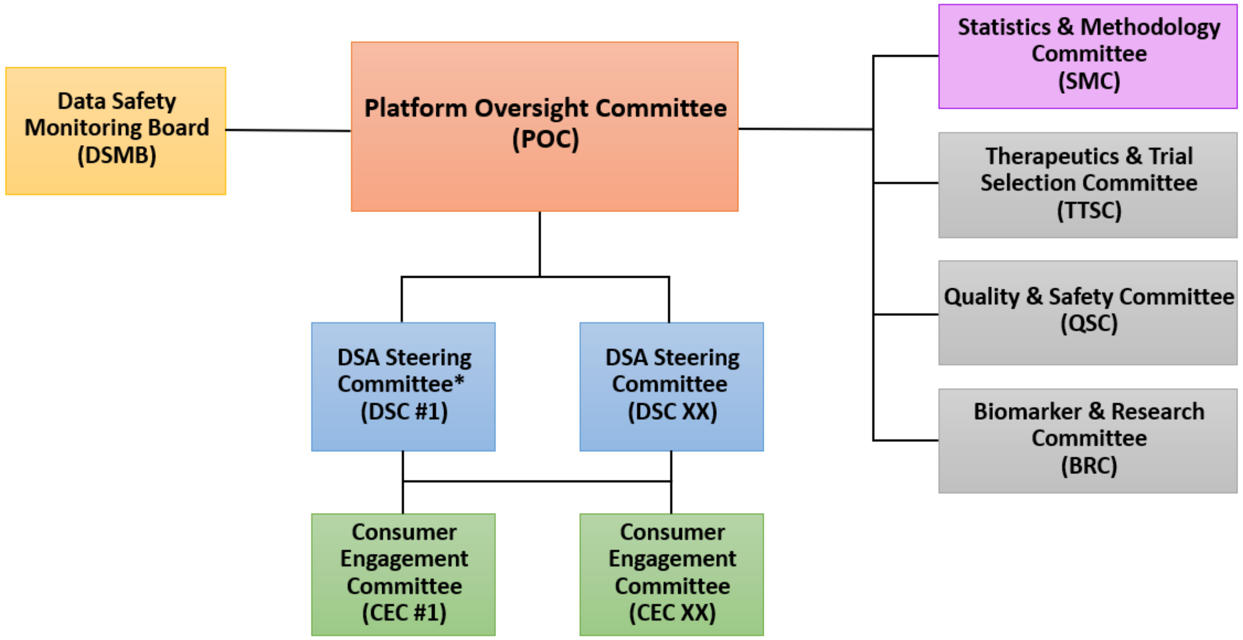

*DSA, Domain-Specific Appendix

Figure 2: Trial Organigram

4.2 Trial Coordination

The GCC will be based at TGI. The GCC is responsible for the operational delivery of the platform and the oversight of Regional Coordinating Centres (RCCs). The GCC will report to the POC on trial operations, logistics, finance, and work with the POC to manage quality and safety within the trial.

Each region will have a RCC that takes primary responsibility for managing participating sites and for managing regional trial activities.

5 BACKGROUND & RATIONALE

5.1 Introduction and Setting

5.1.1 Burden of Chronic Kidney Disease

Chronic kidney disease (CKD) is estimated to affect > 800 million individuals globally. It is a major global concern due to its risk for kidney failure, cardiovascular events, increased disability, and premature death, and is projected to rise to the 5th most common cause of death by 2040(1). This is in stark contrast to other prevalent non-communicable diseases, such as cardiovascular disease and cancer, which have seen a decline in mortality rate(2). CKD is also known to be an independent risk factor for cardiovascular death. In 2017, CKD was associated with 1.2 million deaths worldwide, and a further 1.4 million deaths attributed to cardiovascular disease were implicated by reduced kidney function(3).

CKD is diagnosed by identifying either a reduction in kidney function using creatinine levels in the blood to calculate an estimated glomerular filtration rate (eGFR; normal eGFR>90mL/min/1.73m^2) and/or the presence of albuminuria or proteinuria. The high prevalence is driven by the rising incidence of diabetes and hypertension, the two main causes of CKD, and an ageing population. A multitude of other pathologies, many of which are not completely understood, also lead to the development of CKD albeit with different mechanisms, with the risk of poor outcomes increasing as kidney function declines (4, 5).

The impact of CKD, its complications and sequalae are profound at individual, societal and global levels. CKD is a progressive disease that leads to a loss of kidney function over time and in a proportion of patients leads to kidney failure. Those that experience kidney failure either die or require life-sustaining kidney replacement therapy (KRT), such as dialysis or transplantation - two of the most expensive therapies available. In 2010, whilst 2.6 million people received KRT worldwide, it is estimated that 4.9 to 9.7 million people required KRT, thus contributing to at least 2.3 million premature deaths due to inability to access treatment(6). The cost of treating Medicare beneficiaries with CKD in the USA in 2019 was $87.2 billion. In Australia, health system expenditure on CKD was estimated to cost $1.8 billion in the 2018-2019 financial year (7). Dialysis and kidney transplantation are estimated to cost over $1 billion each year in Australia (8). This financial burden will continue to increase as the incidence of patients requiring KRT is projected to rise further worldwide (Figure 3)(6). Reducing or slowing the progression of CKD is therefore imperative (9). Modelling has shown that there is a gain of 0.1 productivity-adjusted life years per person for every new case of kidney failure avoided over 10 years (10).

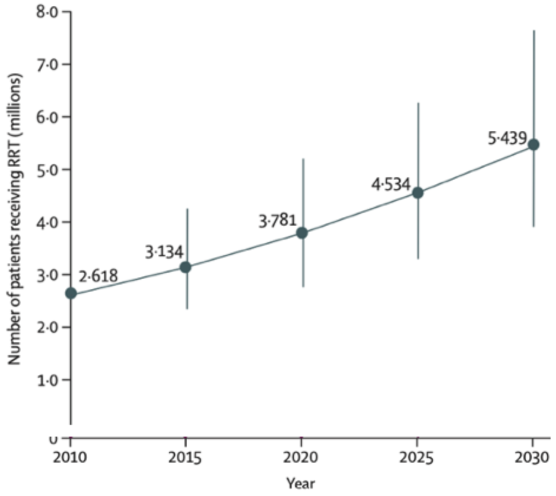

Figure 3: Estimated number of patients undergoing kidney replacement therapy from 2010-2030 globally (Liyanage et al. 2015(6))

5.1.2 Standard of Care for Patients with CKD

The management of CKD currently centres around lifestyle modifications, reducing cardiovascular risk and managing metabolic conditions(11). The two main drivers of CKD are type 2 diabetes mellitus and hypertension, particularly in high- and middle-income countries, with a prevalence of 30-40% in those with diabetes(5). Therefore optimal glycaemic control and strict blood pressure management is also included in standard care(11). Therapeutic agents in CKD have largely been utilized to target a reduction in proteinuria/albuminuria, as this is well recognized to be a predictive marker of poor kidney outcomes, greater cardiovascular risk and increased mortality(5). Specifically, inhibition of the renin-angiotensin system (RAS) with angiotensin-converting enzyme inhibitors (ACEi) and angiotensin receptor blockers (ARB) has been the pillar of CKD treatment for decades.

Whilst ACEi and ARBs have had a profound impact on the trajectory of kidney disease, these major studies were conducted over 2 decades ago, and the prevalence and burden of CKD remains high and they do not entirely ameliorate the risk of CKD.

Over the last decade, sodium-glucose cotransporter-2 inhibitors (SGLT2i) have emerged as an additional drug class that reduces the risk of kidney failure and albuminuria. A meta-analysis of 9 multicentre randomised controlled trials (RCTs) including 25,749 participants with an eGFR <60 mL/min/1.73m2 and 12,863 participants with a urine albumin to creatinine ratio (uACR) >300 mg/g has shown that, compared with placebo, SGLT2i reduced the risk of major adverse kidney events (doubling of serum creatinine, sustained 40% decline in eGFR, kidney failure or death due to kidney disease) by 30% in those with an eGFR <60 mL/min/1.73m2 (hazard ratio (HR) 0.70; 95% confidence interval (CI) 0.58-0.83) and by 43% in those with uACR >300 mg/g (HR 0.57; 94% CI 0.48-0.67) (9). SGLT2i are now widely accepted as standard of care in people with CKD and albuminuria (10) and are also recommended by the National Institute for Health and Care Excellence (NICE) guidelines as a treatment option for people with CKD. They are recommended as an add-on and are increasingly considered standard of care.

It is anticipated that as new therapies are discovered and incorporated into therapeutic regimens, and clinical guidelines, the definitions of standard of care will be updated. Additionally, it is possible that

standard of care could be different across countries. As such, we anticipate that CAPTIVATE will collect data on standard of care at entry into the platform trial and during participation in each DSA. All data on concomitant medications will also be collected.

5.1.3 Study Rationale

Need for more therapies

The risk of kidney failure remains high in a significant proportion of patients, with those with persistently high albuminuria experiencing the greatest risk. Recent advances have led to the discovery of the kidney protective benefits of both existing and novel therapeutic agents such as SGLT2i and mineralocorticoid receptor antagonists (MRA). While these agents reduce the risk of kidney failure, they do not entirely ameliorate this risk, further supporting the investigation and development of new treatments to reduce albuminuria and slow CKD progression (18).

Need to personalise treatment

There is a need to personalise treatments in individual patients with CKD, however, there are minimal head-to-head comparisons between available therapeutic agents nor is there data available on the best combination therapies. As new therapies are identified, data about their benefit over and above standard of care, if there are any synergistic effects and guidance for directing specific types of therapies to specific patients in a timely fashion are essential to rapidly improve outcomes of people with CKD. A structure that permits rapid testing of new agents individually and in combination is essential to increase the options available to patients and health providers for treatment of CKD. Together with these structures, it has been necessary to have valid study outcomes that can be measured rapidly to identify promising therapeutics rather than over several years.

5.1.4 Valid Surrogate Kidney Endpoints

The clinical endpoint used in kidney trials to investigate an intervention's effect on disease progression has conventionally been the halving of eGFR (doubling of serum creatinine) which is a late event in progressive CKD(19). The use of this endpoint results in long follow-up periods of the many thousands of participants in phase II/III clinical trials. The lead up to obtaining marketing approval for successful agents can take up to 15-20 years, with substantial time and resources invested towards therapeutic agents that ultimately fail. eGFR typically declines at a slow rate of approximately 1mL/min/1.73m^2/year, so this endpoint may also not be relevant for those with higher baseline eGFRs or be practical for the study of interventions targeted at earlier stages of kidney disease. Early detection and intervention are critical in CKD, as by the time an individual becomes symptomatic, the disease may be irreversible. Alternative surrogate endpoints including change in albuminuria and eGFR slope have therefore been considered to reduce sample size, shorten trial duration, and reduce trial costs in individuals with earlier disease stages, and ultimately develop effective therapeutic interventions in CKD in an efficient manner to keep up with the rising burden of the disease.

Albuminuria and eGFR are universally validated as predictors for kidney failure independent of each other, with both routinely measured as part of standard of care and utilised in international CKD definitions and prognostic tools(20, 21). Data validating albuminuria reduction and eGFR slope as meaningful endpoints that reliably predict "hard" kidney outcomes of doubling of serum creatinine, kidney failure and death due to kidney failure is an important development as it allows the detection of the early effectiveness of therapy at high statistical power with smaller sample sizes (22, 23). Meta-analysis of trial data shows that a 30% reduction in albuminuria over 6 months is associated with a 27% reduction in risk for the composite of kidney failure, doubling serum creatinine or eGFR <15 mL/min/1.73m^2 over a median follow-up period of 3.4 years(22). This correlation was seen to be stronger in those with a baseline uACR ≥30 mg/g (R^2=0.72).

eGFR is measured as part of routine clinical care at 3-12 monthly intervals and the calculation of eGFR slope (the mean rate of change in GFR from baseline to a designated follow-up time) requires multiple measures and statistical techniques that have been recently developed. Difference in eGFR slope has also shown to have a similar relationship with kidney outcomes in a separate meta-analysis(23). Each 0.75mL/min/1.73m^2/year greater treatment effect was associated with an average 27% lower hazard for the same composite outcome (R^2=0.97) at 3 years. In simulations, eGFR slope increased statistical power compared with the clinical end point of doubling of serum creatinine or kidney failure particularly when the baseline eGFR was high, and allowed for a reduction in trial follow-up from 4-6 years to 2 years and a reduction in sample size by 14-39%(24). From a patient perspective, kidney function has been identified as the most important outcome lending further credibility to the use of eGFR slope as a valid marker of kidney function decline as a patient centred outcome in CKD trials(25).

In 2018, the US National Kidney Foundation (NKF), US Food and Drug Administration (FDA) and European Medicines Agency (EMA) cosponsored a collaborative scientific workshop to evaluate the change in albuminuria and eGFR as surrogate endpoints for clinical trials in early stages of CKD(26). The joint recommendations based on expert review of the latest trial data and meta-analysis included:

- For using albuminuria
    - Early albuminuria reduction can be a "reasonably likely or valid surrogate end point" in phase III RCTs of participants with moderate to severe albuminuria
    - The treatment effect can be detected within 6 months
    - A 21-27% reduction in uACR provides a 97.5% positive predictive value for a non-zero benefit on clinical outcome in participants with a baseline uACR ≥30mg/g
- For using eGFR slope
    - eGFR slope can be a "valid surrogate end point" in phase III RCTs
    - The treatment effect can be detected within 2 years
    - A eGFR slope reduction of 0.5-1.0 mL/min/1.73m2 per year provides a 97.5% positive predictive value for a non-zero benefit on clinical outcome

The use of albuminuria reduction as a surrogate end point has recently led to accelerated FDA approval of novel therapeutic drugs in kidney disease such as targeted-release budesonide and Sparsentan in IgA nephropathy, whilst awaiting longer-term follow up of the confirmatory eGFR slope endpoint(27-30). This has therefore been an important and highly anticipated step in the evolution of CKD trials and drug discovery.

5.2 Adaptive Platform Trials

Innovative trial design has been identified as a core element to advance the therapeutic armamentarium of CKD (34, 35). An adaptive platform trial allows the evaluation of multiple interventions in a single disease within a standing single trial infrastructure with no fixed end date allowing for efficient and accelerated drug development (36, 37). Platform trials are well established in areas such as oncology and infectious diseases (e.g. the RECOVERY trial), however they are yet to be implemented in kidney disease (38). This proposal aims to bring this research paradigm shift to the field of nephrology. The CAPTIVATE trial will establish the first investigator-initiated platform trial for people with CKD, which will enable future trials in CKD to be dramatically cheaper and more efficient as the study design permits the rapid addition of new interventions of interest to existing DSAs and/or addition of new DSAs of interest.

An adaptive platform trial is an extension to the adaptive design whereby multiple trial interventions can be assessed simultaneously in a trial using a core protocol to find the most effective treatment [20]. This

design allows for ineffective agents to be removed from the trial on the basis of a pre-defined decision rules, and for new interventions to be added into the trial through new or existing trial DSAs when available. By allowing simultaneous participation in multiple DSAs, interactions between interventions in different DSAs can be evaluated. The use of response adaptive randomisation is common in platform trials. This allows the randomisation ratio to change over time based on accruing data and maximises the chances of randomisation to an optimal intervention and reduces the chances of randomisation to a futile intervention.

6 OBJECTIVES

6.1 Primary Objective

To determine investigational agents or combinations of agents that reduce the rate of eGFR decline (slow progression of CKD), compared to placebo, in patients with chronic kidney disease receiving standard of care therapy.

6.2 Secondary Objectives

Secondary objectives are to determine the effect of the interventions on:

1. Change in albuminuria between randomisation and 24 weeks
2. Proportion of participants experiencing a 40% eGFR decline, and proportion of participants developing kidney failure (defined as eGFR <15 mL/min/1.73m2 or chronic kidney replacement therapy start) at 108 weeks
3. Time to ≥40% eGFR decline from randomisation or kidney failure
4. All-cause mortality at 108 weeks
5. Proportion of participants experiencing one of more cardiovascular events (cardiovascular death, hospitalised heart failure, myocardial infarction, stroke) between randomisation and 108 weeks
6. Time to first occurrence of a cardiovascular event
7. Safety and tolerability of the intervention
8. Change in quality of life measured using the Quality of Life Impact Survey for Kidney Disease (QDIS-CKD) at 6-monthly intervals from randomisation to week 108

6.3 Tertiary Objectives

Tertiary objectives are to determine the effect of the interventions on:

1. All-cause mortality at 5 years and at subsequent 5-year intervals
2. Proportion of participants who have developed kidney failure at 5 years and at subsequent 5-year intervals, as defined by requirement for chronic kidney replacement therapy or kidney transplant surgery

6.4 Exploratory Objectives

Exploratory objectives are to determine the effect of the interventions on:

1. Health care resource utilisation and costs
2. Composite of mortality, kidney failure, eGFR decline and other endpoints using a win ratio approach.

6.5 DSA-Specific Objectives

Domain-specific objectives will be outlined in the respective DSA.

7 TRIAL DESIGN

7.1 Overview

CAPTIVATE is an innovative multi-centre, multi-arm, Phase III, placebo-controlled, parallel group, adaptive platform randomised controlled trial (RCT) in patients with CKD. Where possible, participants in CAPTIVATE will be actively followed up for 108 weeks post-randomisation, and passively followed up until death. The primary outcome of eGFR slope will be calculated using values obtained from randomisation to week 108.

There is a plan for a pipeline of various therapeutic agents to be studied in the future which will be added as DSAs under the overarching core protocol. Interventions will be evaluated in participants receiving standard of care therapy. DSAs will be implemented in accordance with the core protocol, however, they may have additional characteristics or design features that will be described in the relevant DSA. Examples of trial design features that may vary between DSAs include, but are not limited to: (i) number of active treatment arms, (ii) randomisation strategy (e.g. fixed randomisation as in traditional RCTs or response adaptive randomisation), and (iii) inclusion of rescue randomisation where a participant is not responding to their first-line treatment.

Participants will be able to participate concurrently and also sequentially in more than one DSA, provided that they meet DSA eligibility criteria. Decisions regarding co-enrolment in other clinical trials will be made on a trial-by-trial basis by the Chief Investigators.

CAPTIVATE is intended to be perpetual and will continue to operate until there are no open DSAs, and no participants in follow-up.

7.2 Trial Setting

The trial will be conducted at kidney and endocrinology units/practices globally. CAPTIVATE will collaborate with trials networks globally who are interested.

7.3 Platform Eligibility Criteria

To be eligible to participate in CAPTIVATE, potential participants must fulfil all of the inclusion criteria and none of the exclusion criteria.

7.3.1 Inclusion Criteria

Potential participants must satisfy all of the following:

1. Age ≥ 18 years
2. Known chronic kidney disease from any cause (eGFR ≥25 mL/min/1.73m2)
3. Currently receiving standard of care treatment according to treating physician
4. Eligible for randomisation in at least one recruiting domain-specific appendix
5. Participant and treating physician are willing and able to perform trial procedures

7.3.2 Exclusion Criteria

Potential participants must have none of the following:

1. Currently receiving maintenance dialysis
2. Planned to commence kidney replacement therapy or kidney transplant surgery in next 6 months
3. Life expectancy less than 6 months

7.3.3 Domain-Specific Criteria

Domain-specific inclusion and exclusion criteria will be outlined in the respective DSA.

7.4 Interventions

Interventions include therapeutic treatments that show potential benefit in the area of CKD. The POC is responsible for approving the addition of new interventions to CAPTIVATE based on pre-specified intervention-assessment criteria. Proposed interventions will be assessed on a range of criteria, including:

1. Sound rationale and robust hypothesis that supports the need to assess the intervention within CAPTIVATE setting
2. Intervention is sufficiently differentiated from other interventions currently or previously assessed in CAPTIVATE
3. Clear path for trial results for the intervention to be translated into improved clinical care or public health, with results being still relevant at the time of data maturity
4. No data anticipated to be available that will impact on the validity and feasibility of the intervention while it is being trialled in CAPTIVATE
5. Capacity to integrate the intervention into CAPTIVATE
6. Recruitment to intervention is feasible with respect to sample size and eligibility criteria
7. Acquisition and delivery of the intervention to participating sites is feasible
8. Funding is available to support the trial intervention from relevant industry partner or from a relevant research grant.

The POC will have sole decision-making responsibility for the early stopping of any interventions. Decisions will be informed by recommendations from the DSMB.

Information specific to each intervention is detailed in DSAs. If a run-in period is required for an intervention between recruitment into the DSA and randomisation, this will be described in the relevant DSA. Run-in periods can be used to exclude participants from subsequent randomisation who are non-adherent to protocol or intolerant to treatment.

The DSC will evaluate the suitability of DSAs for specific countries or regions. The default position within CAPTIVATE is that all interventions within the DSA must be feasible and suitable in the region in order for the DSA to be made available. All DSAs that are available in the country or region will be offered to each individual site. Principal investigators at individual sites can select the DSAs that will be available at their site. Should a site choose to participate in a DSA, all interventions within the DSA must be made available at the site unless exemptions are specified within the DSA. Unless specified within the relevant DSA, participants will be randomised to receive one intervention within each DSA.

Should a DSA permit selected interventions to be omitted from either individual regions or sites, details will be specified in the relevant DSA.

Participants must be receiving standard of care therapy according to their treating physician at entry into the trial and during their participation in any DSA. Further details about standard of care treatment in each country will be provided in a Standard of Care Appendix.

7.5 Outcomes

The default position within CAPTIVATE is that all outcomes in the core protocol will apply to each domain. It is recognised, however, that this might not always be feasible. Should a domain depart from the core outcomes, this will be detailed in the relevant DSA.

Site assessed and reported platform outcomes will be used for CAPTIVATE. The primary outcome of eGFR slope is not subject to ascertainment bias. Where possible, trial personnel at site who are blinded to treatment allocation status, will conduct study follow-up visits.

7.5.1 Primary Outcome

The primary outcome is the eGFR slope calculated using eGFR values from randomisation to week 108.

eGFR slope measures the rate of eGFR decline. eGFR measurements will be derived using serum creatinine values applied to the CKD-EPI equation. All blood tests will be performed at local laboratories.

eGFR values are collected at all study visits. eGFR at randomisation (baseline) is the average of two pre-randomisation values comprising:

i. eGFR result from randomisation visit, and
ii. eGFR result from the screening visit.

The Statistical Analysis Appendix will detail how the eGFR slope is calculated, including how missing data values will be managed.

7.5.2 Secondary Outcomes

Secondary outcomes that will be evaluated are:

1. Change in albuminuria as measured by uACR (or uPCR if uACR unavailable) between randomisation and 24 weeks, measured as a continuous variable.
2. Composite outcome of proportion of participants experiencing a 40% eGFR decline between randomisation and 108 weeks, and proportion of participants developing kidney failure (defined as eGFR <15 mL/min/1.73m2 or chronic kidney replacement therapy start†) at 108 weeks.
3. Time to a composite outcome of ≥40% eGFR decline from randomisation or kidney failure
4. All-cause mortality at 108 weeks
5. Proportion of participants experiencing one or more cardiovascular events (cardiovascular death, hospitalised heart failure, myocardial infarction, stroke)* between randomisation and 108 weeks
6. Time to first occurrence of a cardiovascular event
7. Safety and tolerability of treatment
8. Change in quality of life measured using the Quality of Life Impact Survey for Kidney Disease (QDIS-CKD) at 6-monthly intervals from randomisation to week 108.

†Chronic kidney replacement therapy is defined as:

- Receipt of kidney replacement therapy for more than 3 months, or
- Deemed to be chronic kidney replacement therapy by the participant's treating physician.

*Clinical definitions for cardiovascular events are provided in Appendix 16.1.

uACR values are collected at all study visits. uACR at randomisation (baseline) is the average of 2 pre-randomisation values comprising:

i. uACR result from randomisation visit, and

ii. uACR result from the screening visit.

The interim analysis at 24 weeks will use the change in albuminuria as an intermediate outcome to assess likely treatment efficacy. Details of any further interim analyses will be provided in the Statistical Analysis Appendix.

The safety of treatment will be assessed by incidence and rates of adverse events (AEs) and serious AEs. See Section 11 for the definition of and reporting requirements for AEs.

Tolerability of treatment is defined as the time from commencement of the study treatment until interruption of treatment due to toxicity. Interruption of treatment includes treatment discontinuation or a pause in treatment. Toxicity is defined as a treatment-related AE of any grade that leads to the interruption of study treatment. An AE is deemed to be due to treatment if the causal relationship between the event and the study treatment is judged by the Investigator to be definitely, probably or possibly related.

The Quality of Life Impact Survey for Kidney Disease (QDIS-CKD) is a short, 7-item instrument that captures patient-reported outcomes via multiple functional health and wellbeing domains (role and social functioning, fatigue, psychologic distress, cognitive functioning, physical functioning) and overall quality of life. Assessment of such patient-reported outcomes is important as CKD and its treatment can have considerable impacts on patient experiences and well-being. The QDIS-CKD was developed by the John Ware Research Group, Inc who kindly shares the instrument for use in the CAPTIVATE trial. The QDIS-CKD showed better discrimination than the generic SF-12 Health Survey and better validity than the Kidney Disease Quality of Life-36 (KDQOL-36) instrument for clinical status, clinician assessment of change for CKD-specific tests and number of comorbidities in patients with CKD (39). See Appendix 16.2 for the QDIS-CKD questionnaire.

7.5.3 Tertiary Outcomes

Tertiary outcomes that will be evaluated are:

1. All-cause mortality assessed at 5 years and at subsequent 5-year intervals
2. Proportion of participants who have developed kidney failure assessed at 5 years and at subsequent 5-year intervals, as defined by requirement for chronic kidney replacement therapy or kidney transplant surgery

7.5.4 Exploratory Outcomes

Health care resource utilisation and costs will be evaluated as an exploratory outcome using information obtained from data linkage to other datasets, such as routinely collected administrative data. This will only occur should linkage to these datasets be feasible.

Additionally, win ratios will be considered as exploratory outcomes. Composite outcomes that will be evaluated with a win ratio approach may include, but are not limited to:

- All-cause mortality
- Proportion of participants developing kidney failure
- Proportion of participants experiencing a 40% eGFR decline
- Rate of eGFR decline

A win ratio approach accounts for clinical priorities.

8 TRIAL CONDUCT

8.1 Consent

8.1.1 Trial Consent

Trained research staff will introduce the trial and provide Participant Information Sheet and Consent Forms (PISCFs) to patients. A Core PISCF will describe in detail the information needed for a patient to decide whether they would like to participate in the platform. This information includes; the exact nature of the trial, the implications and constraints of the protocol and any risks involved in taking part. In addition, DSA-specific PISCFs will be provided to the patient for all active DSAs for which the patient is clinically eligible. Information in the DSA-specific PISCFs includes the known side effects of the interventions within the DSA, the exact nature of the DSA, the constraints of the DSA and any risks involved in taking part in the DSA.

Trial staff will discuss the platform and relevant DSAs with the patient in detail. Patients will be given sufficient time to consider the information as per local practice and be able to have an informed discussion with their relevant medical professionals. Patients will be encouraged to discuss their potential participation in the trial with family members and close friends.

All participants must provide written informed consent or eConsent for the Core platform and for individual DSAs that they consent for, prior to the commencement of any trial-related procedures. The person who obtained the consent must be suitably qualified and experienced and have been authorised to do so by the site Principal Investigator. Written informed consent may be replaced by electronic signature on an eConsent form in countries where eConsent is permitted by local regulatory requirements. Further details will be provided in individual CSAs.   A copy of the signed consent form will be given to the participant.

If the participant is unable to read, oral presentation and explanation of the PISCF to be supplied to the participant must take place in the presence of an impartial witness. Consent must be confirmed at the time of consent orally and by the personally dated signature of the participant or by a local legally recognised alternative (e.g. the participant's thumbprint or mark). The witness and the person conducting the informed consent discussions must also sign and personally date the consent document.

In countries where data linkage will be conducted, specific consent for data linkage will be sought from participants. Consent for data linkage is an optional component of the trial.

The medical record must include a statement that written informed consent or eConsent was obtained before the participant was enrolled in the study and the date of consent.

8.1.2 Withdrawal of Consent

Participants have the right to withdraw their consent to participate in the research at any time, and it will not affect their rights as a patient, including the health care they receive outside of the trial. Participants may withdraw from the trial entirely or from one or more DSAs. To document withdrawal from the trial entirely, the participant and a trained research staff member (or just the trained research staff member, where verbal withdrawal is given) can complete a Withdrawal Form, however participants can also withdraw verbally from the trial without needing to give a reason. The ability for the research staff to continue limited data collection via passive follow-up should be discussed. In the majority of cases,

participants give permission for passive follow-up and data collection to continue from hospital records. In these cases, this does not constitute withdrawal from the trial.

All communication around the withdrawal of a participant should be documented within the participant's records. If a participant does not give permission for passive follow-up, this constitutes withdrawal from the trial and should ideally be documented on the Withdrawal Form and in the participant's medical records. For participants who do not give permission for passive follow-up, data collected up to the point of participant withdrawal will be used in the analyses, but no further data will be collected on these participants.

8.2 Participant Schedule

The participant schedule is designed to allow study visits and assessments to be conducted during routine visits for clinical care where possible. Patients with CKD have regular visits with their treating physician as part of their routine clinical care. The frequency of these visits depends on local practices and patient factors, but their frequency ranges from 3 months to 12 months.

The default participant schedule for the CAPTIVATE platform is detailed in this section of the core protocol. The goal of the trial is for domains to conduct all the procedures in this core participant schedule, such that the data variables listed in this Core Protocol will be collected for all DSAs. It is recognised, however, that this might not always be feasible. Should a domain depart from the core participant schedule, this will be detailed in the relevant DSA. Possible departures could include omission of specific assessments or visits or change in timepoints for specific assessments. The treatment duration could also depart from the default duration of 104 weeks.

Additional DSA-specific visits and data variables are expected for domains and these are outlined in the relevant DSAs.

Blood tests and urinalysis will be performed at local laboratories. Results from standard of care tests will be used, where available. For urinalysis, a urine sample from the first void for the day should be collected where possible as this is more reliable for monitoring albuminuria than a spot (random) urine sample.

No central laboratory testing is anticipated as part of the core protocol. It is possible that specific DSAs will require blood samples for central laboratory assays and this will be detailed in the relevant DSAs.

Table 1: Core Schedule of Assessments

Assessment	Screening	Randomisation	Active Follow-Up							Early Treatment Discontinuation	Passive Follow-up[5]
**Week**	-4	0	4	12	24	52	78	104 (EOT)	108	4w after early discontinuation (ETDV)	Every 5 years
**Window**	Day -1 to Day -31	-	± 2w	± 4w	± 4w	± 4w	± 4w	± 2w	± 2w	± 2w	± 3m
**Trial consent**	X										
**Demographics**	X										
**Medical history**	X										
**Concomitant medications**	X	X[3]			X	X	X	X	X		
**Blood pressure**	X	X	X	X	X	X	X	X	X		
**Blood tests[1]**	X	X	X[4]	X[4]	X[4]	X[4]	X[4]	X[4]	X	X	
**Urinalysis[2]**	X	X	X[4]	X[4]	X[4]	X[4]	X[4]	X[4]	X	X	
**QDIS-CKD**		X			X	X	X	X	X		
**Trial outcome events**		X	X	X	X	X	X	X	X	X	
**Adverse events**		X	X	X	X	X	X	X	X	X	
**Vital status & Kidney status**											X
**Data Linkage[6]**											X

Abbreviations: EOT = End of Treatment, ETDV = Early Treatment Discontinuation Visit, m = month, QDIS-CKD = Quality of Life Impact Survey for Kidney Disease, w = week

[1] Blood tests: Creatinine, sodium, potassium.

[2] Urinalysis: uACR or uPCR (if uACR unavailable). A urine sample from the first void for the day should be collected where possible.

[3] Concomitant medications do not need to recorded at the Randomisation Visit if there has been no change in medications since concomitant medications were recorded at the Screening Visit and the Screening Visit occurred within 14 days before randomisation.

[4] For all visits from Week 4 to Week 104, the most recent standard of care result for blood tests and urinalysis will be accepted provided that it is dated after the corresponding test result for the previous study visit.

[5] Passive follow-up will continue until all participants are deceased.

[6] Data linkage is optional and will only be performed in countries where linkage is feasible.

8.2.1 Screening (Day -31 to Day -1)

Screening of potential participants will occur through kidney and endocrinology units/practices participating in the trial and specialist referrals to participating Investigators. Participants must meet all the platform inclusion criteria and not meet any exclusion criteria. If a patient is not eligible, they can be re-screened should their situation change.

All assessments for the Screening Visit must be performed within the visit window from Day -31 to Day -1:

- Informed consent for trial
- Demographics, including month and year of birth, age, sex, weight, height, self-reported ethnicity
- Medical history, including cause of kidney disease, present comorbidities, diabetes and cardiovascular history, smoking status
- Record concomitant medications
- Measure blood pressure
- Blood tests, including creatinine, sodium, potassium
- Urinalysis: uACR or uPCR (if uACR unavailable)

For blood tests and urinalysis, results from standard of care tests can be used if they fall within the Screening Visit window.

8.2.2 Randomisation (Day 0)

At the Randomisation Visit (Day 0), the following activities and assessments will be conducted:

- Record concomitant medications†
- Measure blood pressure
- Blood tests, including creatinine, sodium, potassium
- Urinalysis: uACR or uPCR (if uACR unavailable)
- QDIS-CKD
- Record AEs and trial outcome events
- Randomise the participant

† Concomitant medications do not need to be recorded at the Randomisation Visit if there has been no change in medications since concomitant medications were recorded at the Screening Visit and the Screening Visit occurred within 14 days before randomisation.

Blood test and urinalysis must be performed on the day of the randomisation visit so that baseline data for trial outcomes are available. Results of these tests do not need to be available for randomisation to occur.

Trained site staff must confirm the participant's eligibility at the time of randomisation. Participants will be randomised between interventions within the DSAs that they are eligible and consent to. Randomisation will be at the participant level and will be performed separately for each DSA for which a participant is eligible. For example, if a patient is eligible for two DSAs, they will be randomised twice, once in each DSA.

Randomisation will be conducted through a password-protected, secure website using a central, computer-based randomisation program. This centralised randomisation will ensure that allocation concealment is maintained.

The allocated study intervention should start as soon as possible after randomisation, and continue until week 104, or until a trial stopping point is reached. Further details about treatment-specific procedures will be outlined in the relevant DSA.

8.2.3 Active Follow-Up

Participants will have an active follow-up period of 108 weeks following randomisation. Study assessments at each visit are standardised across each DSA. Assessments at each follow-up visit include:

- Record concomitant medications (not performed at Weeks 4 and 12)
- Measure blood pressure
- Blood tests, including creatinine, sodium, potassium†
- Urinalysis: uACR or uPCR (if uACR unavailable)†
- QDIS-CKD (not performed at Weeks 4 and 12)
- Record AEs and trial outcome events

† For all active follow-up visits from Week 4 to Week 104, the most recent standard of care result for blood tests and urinalysis will be accepted, provided that it is dated after the corresponding test result for the previous study visit. This will facilitate the use of standard of care tests.
For the Week 108 visit, blood test and urinalysis must be performed within the ±2 week visit window.

If a participant expresses a wish not to attend the follow-up visits in person, this will be discussed with the participant with the following options for contact: phone contact, follow-up via medical record review, family members, data linkage and/or other treating physicians. Every effort will be made to determine survival status and trial outcomes. If required, blood test request forms can be sent to participants. Consent forms at study entry include consent to the above-mentioned options for contact and all other legal means to determine vital status.

8.2.4 End of Treatment

The randomised treatment will stop at the End of Treatment (EOT) visit at week 104, or sooner in the event of a stopping rule being met. For participants in an intervention that meets a stopping rule, an EOT visit for the intervention is to be scheduled within 30 days of being notified of the stopping rule being met and treatment being discontinued.

8.2.5 Final Visit

A final study visit for the intervention will occur 4 weeks after the EOT visit. If a stopping rule was not met, the final visit occurs at week 108. The final follow-up visit marks the end of the active follow-up period.

If a stopping rule was met, the final visit does not occur and the EOT is the last study visit for the successful or futile intervention.

8.2.6 Early Treatment Discontinuation Visit (4 weeks after early discontinuation of treatment)

The Early Treatment Discontinuation Visit (ETDV) is only performed if the participant permanently discontinues the randomised treatment before the EOT visit at week 104, and this is not due to a stopping rule being met. Where practical and feasible, an ETDV will be performed 4 weeks (±2 weeks) after the

final administration of the randomised treatment. It is important for blood tests to be performed within this visit window.

Assessments at the ETDV include:

- Blood tests, including creatinine, sodium, potassium
- Urinalysis: uACR or uPCR (if uACR unavailable)
- Record AEs and trial outcome events

If the ETDV coincides with a scheduled study follow-up visit, the scheduled follow-up visit will replace the ETDV. Options for performing the visit remotely as per all active follow-up visits apply to the ETDV.

After the ETDV, participants will continue study follow-up visits as per the Schedule of Assessments to ensure that data are collected for survival status and trial outcomes.

8.2.7 Passive Follow-Up

Passive follow-up will be performed at 5 years and at subsequent 5-year intervals after entry into the CAPTIVATE platform trial by site staff via medical record review, and/or via data linkage for participants who consent to this and where this is an available option and resources allow. Passive follow-up will end only when all participants are deceased. The following data will be collected:

- Vital status
- Kidney status: Whether the participant requires kidney replacement therapy, or has undergone or requires kidney transplant surgery.
- Additional data that may be obtained from data linkage is detailed in Section 8.3.

8.2.8 Site Transfer

All participants will remain in the trial until the end of the study even if their care is transferred to another hospital or facility. If the other hospital or facility is a CAPTIVATE site, all trial-related procedures will also be transferred to the new hospital or facility. If the other hospital or facility is not a CAPTIVATE site, the original hospital or facility should continue to conduct all trial-related procedures, including dispensing the study intervention, where possible.

8.2.9 Loss to Follow-Up

Every effort will be made to contact participants who are deemed 'lost to follow-up' and who have not withdrawn consent for further data collection. Consent forms at study entry include consent to alternate options for contact (phone contact, follow-up via medical record review, family members, and/or other treating physicians), data linkage and all other legal means to determine vital status and trial outcomes.

8.2.10 Participant Discontinuation from Trial

Trial participants may be discontinued from the platform entirely or from one or more DSA according to predefined criteria for discontinuation. Criteria for discontinuation specific to each DSA, if applicable, are specified in the relevant DSA.

Criteria for discontinuation from the CAPTIVATE platform entirely include:

1. The treating physician considers continued participation in CAPTIVATE is not in the best interests of the participant.
2. The participant withdraws consent for participation in the entire platform trial.

In the case of discontinuation, the reasons for discontinuation from the trial will be documented. Data collected up to the point of discontinuation will be used in analyses. Participants will be treated according to standard of care treatment following trial discontinuation. Participants who are withdrawn or discontinued from the trial will not be replaced.

8.3 Data Linkage

To facilitate the evaluation of the tertiary and exploratory objectives, data linkage will be conducted in countries where this is feasible and resource allow. The CAPTIVATE trial will seek to link participant data collected within the trial to other datasets such as routinely collected administrative data, including death registries, hospital admission and emergency department datasets, and datasets that record the use of kidney replacement therapy, the dispensing of prescription medicines and health service utilisation. Linked data will be extracted to obtain vital status, kidney status and health care utilisation data where feasible.

The evaluation of health care resource utilisation and costs is only possible through the use of linked data. Healthcare utilisation data of interest include inpatient days, outpatient visits, physician visits and data on concomitant medications. Costs will be calculated based on representative regional unit costs at the point of analysis. Economic evaluation will focus on interventions with a significant efficacy outcome.

Further details about data linkage and the use of linked data will be described in the relevant CSAs.

8.4 Management of Pandemics & Exceptional Circumstances

Restrictions on movement due to pandemics or other exceptional circumstances may prevent participants from attending clinic visits. These situations could include COVID-19 travel restrictions and natural disasters. To minimise the impact of these disruptions, all study visits may be conducted remotely if COVID-19 or other exceptional circumstances prevent the visit from being performed in-person. The participant can be contacted via video conference, telehealth or telephone. Informed consent may be obtained remotely via eConsent. As many of the study procedures as possible should be conducted at each remote visit. It is important that safety assessments and the recording of trial outcomes events are performed. Participants can self-report blood pressure measurements provided that they have access to a blood pressure monitor. If the study intervention needs to be dispensed, the site can arrange delivery to the participant according to local processes. This could include the use of a courier to deliver the study intervention to the participant's home.

To provide flexibility, sites can use any method that is locally available to conduct the trial provided that these methods meet local regulatory requirements.

8.5 Blinding & Unblinding

The default position within CAPTIVATE is that treatment allocations determined by randomisation will be provided on a double-blinded basis. Blinding, however, may not always be feasible particularly for behavioural or device-related interventions. Details related to the blinding or open-label status of interventions will be specified in DSAs.

Where a DSA is double-blinded, the following principles will be adhered to. Participants, site personnel, investigators and trial statisticians will remain blinded to the treatment from the time of randomisation until database lock of the comparisons for which that participant is contributing data to. Randomisation data will only be accessible to: (1) data managers who work on the randomisation and drug management

system, (2) unblinded statistician(s) involved with the response adaptive randomisation, (3) the unblinded statistician who prepares reports for the DSMB, and (4) pharmacovigilance personnel involved with submitting unblinded safety reports to regulatory authorities.

Emergency unblinding should only be undertaken when it is essential for participant safety and will only be provided when knowledge of the treatment will influence the participant's management in a significant fashion. Detailed information regarding the unblinding procedure will be provided in the Manual of Operations. In case of unblinding, only those individuals who are required to know the treatment allocation should be given this information. All others must remain blinded, including the participant.

At the end of an interventional comparison within a DSA and acceptance for publication of the manuscript reporting the main results, all participants enrolled within the reported data and their treating physician can be un-blinded to that particular intervention.

8.6 Treatment of CKD

8.6.1 Standard of Care

All participants will receive standard of care treatment for their CKD as per local clinical guidelines. For the purposes of CAPTIVATE, standard of care will be defined as treatment provided outside of the protocol by the treating physician in direct response to the CKD diagnosis or progression. Standard of care for this patient group may vary considerably across regions. The Standard of Care Appendix contains further details of standard of care treatment.

8.6.2 Protocol Study Interventions

Protocol study interventions include any protocol-mandated treatments. Protocol study interventions will be detailed within the DSAs accompanying this Core Protocol. As CAPTIVATE is designed to investigate a range of different therapeutic approaches within the same trial infrastructure, study interventions may be pharmacotherapeutic, involve a medical device, or be another modality of therapy.

The protocol study intervention can be stopped at the participant or treating physician's discretion at any time without termination of their participation in the trial.

8.7 DSA Closure

For the purpose of complying with local regulations, DSAs will be considered 'closed' when active follow-up has ceased in all participants within the DSA, and the DSA analyses have been performed and appropriately reported.

Following DSA closure, CRFs, clinical notes and administrative documentation must be kept in a secure location. It is permissible to archive this information providing that it can be made accessible and available to the competent or equivalent authorities, the Sponsor, and other delegated authorities with suitable notice as the data may be subject to audit or inspection from any of the above.

Should an intervention within a DSA be shown to be efficacious, it may be possible for participants to access the treatment following DSA closure. If access to the treatment can be provided following DSA closure, this will be detailed in specific DSAs. If DSA results lead to changes in clinical guidelines, access may also be possible via standard of care treatment.

8.8 End of Trial

CAPTIVATE will continue to operate until there are no open DSAs, and no participants in follow-up. Passive follow-up and thus the operation of CAPTIVATE continues until all participants are deceased.

8.9 Post-Trial Care

The trial has no responsibility for the ongoing management or care of participants following the cessation of all trial specified interventions. Participants will revert to standard of care treatment under the care of their treating physician.

9 DATA MANAGEMENT

9.1 Data Collection & Handling

Data will be collected as per the schedule of assessments and will be kept standardised as much as possible across all DSAs. All trial data, except for linked data, will be captured electronically using a web-based Electronic Data Capture (EDC) system with trial specific electronic Case Report Forms (eCRFs). Linked data will be stored separately and merged to facilitate an analysis.

The Investigator should ensure the accuracy, completeness and timeliness of the data reported in the eCRF and in all required reports, including safety documentation. Information recorded in the eCRF must be collected from source documents, which for CAPTIVATE will primarily be from the participant medical records. An electronic audit trail will maintain a record of initial entries and changes made; reasons for change; time and date of entry; and username of person who made the change.

The platform will utilise a software as a service relationship with an EDC provider and all data will be centrally stored on TGI servers. Data will be securely stored and accessed only by approved study team personnel. Authority over access will be managed by the POC, with delegation to the GCC.

A trial identification number will be allocated to each participant entered into the eCRF. The trial ID will be the primary way in which the participant will be identified and should be used in all correspondence

Further details about data handling will be described in the CAPTIVATE Data Management Plan.

9.1.1 Archiving

All trial data will be archived when appropriate in a secure environment that can be retrieved for audit if required and stored for the appropriate regulatory required period. Guidance will be sought from site institutional policies in order to adhere to these requirements.

9.2 Data Sharing

9.2.1 Data Ownership

All data collected as part of CAPTIVATE will be owned by the Sponsor under the custodianship of the POC. All trial data will be retained for 25 years after the end of the trial or according to local regulation. As the trial is intended to be perpetual, this may result in trial data being retained indefinitely.

The POC will be delegated the responsibility of deciding on behalf of the Sponsor how and when the trial data should be disseminated or released following request.

9.2.2 Data Dissemination

At the conclusion of each DSA, results will be communicated to participants, investigators, trial staff, and the consumer and scientific communities via a variety of media including a newsletter, publication in a peer reviewed journal, conference presentation and the Sponsor's websites. Manuscripts and abstracts will be prepared by the corresponding DSC. If there are interactions between DSAs, the DSCs for all affected DSAs will be responsible for preparing publications. All manuscripts and abstracts reporting trial results must be submitted to and approved by the POC before submission.

Following the primary publication, a number of subsequent publications may arise from analysis of secondary, tertiary and exploratory outcomes, sub-studies yet to be developed, or from exploratory analysis of the trial data set. The number and frequency of these publications will be informed by the trial outputs. Any CAPTIVATE investigator can submit publication proposals to the relevant DSC for consideration. If the proposal relates to the platform trial as a whole, rather than to specific DSAs, the publication proposal can be submitted to the POC.

All publications will be subject to POC oversight and consistent with the International Committee of Medical Journal Editors Recommendations for the Conduct, Reporting, Editing and Publication of Scholarly Work in Medical Journals. Site investigators and research coordinators at participating sites will be acknowledged by their names being listed as collaborators.

Local investigators must not disseminate data including interim or final results via publication, or presentation, without the written consent and support from the POC.

Full details of processes governing publications and data dissemination will be described in the CAPTIVATE publication policy.

9.2.3 Data Release Requests

Data sets specific to a comparison will be made available by the Sponsor to researchers within the CAPTIVATE trial for analysis of sub-studies and country-specific outcomes after the primary manuscript for that comparison has been accepted for publication, at the discretion of the POC.

Trial data will be made available as a resource for future unspecified research, subject to any prior contractual obligations. Researchers wishing to gain access to the study resources will be required to submit an application for review by the POC, including a detailed project description, a list of data requested, and evidence of ethical approval. Assessments of proposals will be based on sound science, benefit-risk balancing and research team expertise. De-identified data extracts will be made available to approved proposals, and will not include data obtained from data linkage with local registries.

10 QUALITY MANAGEMENT

10.1 Site Management

10.1.1 Selection & Feasibility

The GCC and RCCs will work with the DSCs to identify a number of investigators with interest in participating in the trial. All sites interested in participating in CAPTIVATE will be evaluated for the infrastructure needed and operational feasibility for participating in the trial.

Further details of site selection and feasibility, including a full list of mandatory trial documentation will be described in the CAPTIVATE Quality Management & Monitoring Plan (QMMP).

10.1.2 Training

All sites will undergo a site initiation visit where the core protocol and currently active DSAs will be discussed, and training will be conducted on the procedures and assessments of the trial. In instances where new DSAs are integrated into CAPTIVATE, a domain-specific initiation for each new DSA will be conducted with mandatory attendance from at least one member from each site.

Ongoing training will be provided to site staff on an as-needs basis throughout the trial. Further details of site training will be described in the CAPTIVATE QMMP.

10.1.3 Site Documents

The Investigator must maintain adequate and accurate records to enable the conduct of the study to be fully documented and the study data to be subsequently verified. These documents should be classified into two separate categories (1) Investigator Site File (ISF), and (2) Participant source documents.

The ISF contents will include the protocol/amendments, schedule of assessments, Independent Ethics Committee/Institutional Review Board and governmental approval with correspondence, sample participant information sheet and informed consent, study medication records, staff curriculum vitae and certifications and other essential documents/correspondence.

A source document is a document in which data collected for clinical research is first recorded. Examples of source documents include patient hospital/clinic records, physicians' and nurses' notes, appointment book, original laboratory reports, ECG, X ray, pathology and special assessment reports, participant study medication diary, study medication dispensation log, signed informed consent forms, consultant letters, and subject screening and enrolment logs.

The Investigator must keep all trial-related documentation for at least 25 years after the end of the trial or according to local regulation. Should the Investigator wish to assign the study records to another party or move them to another location, the RCC and GCC must be notified in advance.

10.2 Quality Assurance

CAPTIVATE will be conducted in accordance with the current approved protocol, Good Clinical Practice (GCP), relevant regulations and standard operating procedures. The trial will be monitored by the RCC and GCC, or their delegates. Details of monitoring activities will be described in the CAPTIVATE QMMP. Any domain-specific monitoring issues will be addressed in each DSA.

10.2.1 Central Monitoring

Monitoring will include centralised review of eCRFs and other study documents for protocol compliance, data accuracy and completeness. Data input into the CAPTIVATE database will be checked with a number of validations for missing data points, unusual values and consistency over time. Automatically generated queries and manual queries will be raised for resolution by the investigator or site staff.

10.2.2 On Site Monitoring

Routine monitoring visits to investigational sites will be conducted. The frequency of visits will be determined by each site's rate of recruitment. Should restrictions on movement due to pandemics or other exceptional circumstances prevent on-site monitoring visits, remote monitoring visits will be conducted if feasible and if sites are agreeable. Source documents, investigator site files and other trial-related documents must be made available to the trial monitor. A monitoring report will be prepared following each visit and a follow-up letter sent to the site. Email and telephone communication will supplement site visits.

10.2.3 Audit & Inspection

This trial may be subject to audit or inspection by representatives of the Sponsor, GCC/RCC, the host institution, funding bodies and regulatory authorities. By signing the informed consent form, the participant gives authorised representatives of these bodies direct access to their medical records and trial data.

As soon as the site investigator is notified of a planned inspection by the authorities, they will inform the relevant RCC and GCC, and request their participation in this inspection. Any result and information arising from the inspections by the regulatory authorities will be immediately communicated by the Investigator to the RCC and GCC. The Investigator shall take appropriate measures required by the RCC and GCC to take corrective actions for all areas of opportunity identified during the audit or inspections.

10.3 Protocol Deviations

A protocol deviation is an unanticipated or unintentional departure from the expected conduct of an approved study that is not consistent with the current research protocol or consent document. A protocol deviation may be an omission, addition or change in any procedure described in the protocol.

The site Principal Investigator should not implement any deviation from or changes of the protocol without agreement by the POC and documented approval from the Independent Ethics Committee of the amendment, except where necessary to eliminate an immediate hazard(s) to trial participants. In the event of an emergency intended to eliminate an apparent immediate hazard to participants, the Investigator may implement any medical procedure deemed appropriate, and on report that to the sponsor within 24 hours.

11 SAFETY MANAGEMENT

A summary of the safety management strategy that will be used in CAPTIVATE is outlined in this section. Full details of safety management processes will be described in the CAPTIVATE Safety Management Plan.

The safety reporting requirements for CAPTIVATE are designed to protect the safety and welfare of participants, but avoid the reporting of events that are not informative with respect to determining the safety of the study intervention or study participation. To ensure that any concerning safety signals are promptly detected and managed, several levels of safety monitoring will be implemented. All safety reports that are received from sites will be centrally reviewed by Medical Monitors appointed by the Sponsor. In addition, summary data and line listings for all reported events will be reviewed by Medical Monitors on a monthly basis. The DSA Steering Committee will review safety data at each committee meeting for the domain that they oversee. The POC will review safety data for the entire platform trial at each committee meeting. The highest level of review is the DSMB. The DSMB will review unblinded summary data and line listings at DSMB meetings. Should concerns be raised at any level, these will be escalated to the next level. Escalation can occur outside scheduled reviews and meetings.

11.1 Definitions

11.1.1 Adverse Event

An adverse event (AE) is any untoward medical occurrence in a patient or clinical trial participant administered a study intervention and that does not necessarily have a causal relationship with this treatment. An AE can therefore be any unfavourable and unintended sign (including an abnormal laboratory test finding), symptom, or disease temporally associated with the use of the study intervention, whether or not related to the study intervention.

11.1.2 Adverse Reaction

An adverse reaction (AR) is any AE which is considered to have at least a reasonable possibility of a causal relationship to the study intervention, i.e. a causal relationship cannot be ruled out.

11.1.3 Serious Adverse Event

A serious adverse event (SAE) is any AE that;

- results in death,
- is life-threatening, (i.e. the participant is at risk of death at the time of the event),
  - The term “life-threatening” in the definition of “serious” refers to an event in which the participant was at risk of death at the time of the event; it does not refer to an event which hypothetically might have caused death if it were more severe.
- requires inpatient hospitalisation or prolongation of existing hospitalisation,
- results in persistent or significant disability or incapacity
- is a congenital anomaly or birth defect
- requires medical or surgical intervention to prevent life-threatening illness or injury or permanent impairment, or
- is an important medical event which, in the opinion of the investigator, is likely to become serious if untreated, or as defined in the protocol
  - Important medical events which may not be immediately life-threatening or result in death or hospitalisation but which may jeopardise the participant or may require intervention to prevent one of the listed outcomes in the definition above should also be considered serious.

11.1.4 Serious Adverse Reaction

A serious adverse reaction (SAR) is any SAE which is considered to have at least a reasonable possibility of a causal relationship to the study intervention.

11.1.5 Suspected Unexpected Serious Adverse Reaction

A suspected unexpected serious adverse reaction (SUSAR) is an adverse reaction that is both serious and unexpected. In other words, this is any untoward medical occurrence in a participant, which meets the definition of serious, has a reasonable possibility of a causal relationship to the study intervention, and is unexpected.

11.1.6 Adverse Events of Special interest

An adverse event of special interest (AESI) is an untoward medical occurrence that is considered to be medically significant for the CAPTIVATE trial.

For the purposes of CAPTIVATE, AESIs will comprise of:

1. Hyperkalaemia, defined as any episode of serum potassium > 6.0 mmoL/L
2. Acute kidney injury (AKI) as per KDIGO guideline and defined as any of the following:
    - Increase in Serum Creatinine by ≥0.3 mg/dL (≥26.5 µmol/L) within 48 hours; or
    - Increase in Serum Creatinine to ≥1.5 times baseline, which is known or presumed to have occurred within the prior 7 days; or
    - Urine volume <0.5 mL/kg/h for 6 hours.

Additional events considered to be AESIs and which have the potential to be causally associated with a study intervention will be detailed in specific DSAs.

11.1.7 Significant Safety Issues & Urgent Safety Measures

A significant safety issue (SSI) is defined as a safety issue that could adversely affect the safety of participants or materially impact on the continued ethical acceptability or conduct of the trial. These events may be in addition to AE reports and generally have a consequence related to participant safety within the current study protocol, which thus requires some type of amendment.

An urgent safety measure (USM) is one type of SSI where sponsors or trial investigators act immediately to protect participants from an immediate hazard to their health and safety. USMs are often instigated before the regulatory authorities and ethics committees are notified.

Examples include:

- An SAE that could be associated with the trial procedures and that requires modification of the conduct of the trial
- A hazard to the patient population, such as lack of efficacy of a study intervention used for the treatment of a life-threatening disease
- A major safety finding from a newly completed animal study (such as carcinogenicity)
- A temporary halt/termination of a trial for safety reasons
- Recommendations of the DSMB, where relevant for the safety of participants, such as an increase in frequency or severity of an expected adverse reaction
- Single case events that lead to a USM

11.2 Assessments

11.2.1 Causality

The causal relationship between an AE and the study intervention will be determined by the investigator on the basis of their clinical judgment and the following definitions:

- Definitely related: Event can be fully explained by administration of the study intervention.
- Probably related: Event is most likely to be explained by administration of the study intervention rather than the participant's clinical state or other agents/therapies.
- Possibly related: Event may be explained by administration of the study intervention or by the participant's clinical state or other agents/therapies.
- Unlikely to be related: Event is most likely to be explained by the participant's clinical state or other agents/therapies, rather than the study intervention.
- Not related: Event can be fully explained by the participant's clinical state or other agents/therapies.

An AE is deemed to be treatment related if the causal relationship between the event and the study intervention is judged to be definitely, probably or possibly related.

11.2.2 Expectedness

The expectedness of the event to the study intervention is either:

- Expected, or
- Unexpected

An event is considered unexpected if;

- Not listed in the Reference Safety Information (RSI)
- If severity exceeds what is listed in the RSI
- If frequency exceeds what is listed in the RSI
- If event outcome exceeds what is listed in the RSI

Expectedness will be an assessment based on the toxicities present within the RSI used for each intervention within the platform.

11.3 Pregnancy

The default position within CAPTIVATE is that pregnancy and lactation do not prevent participation in the platform. There may be DSA-specific exclusions for participants who are pregnant or breastfeeding where this is required for safety reasons. Any exclusion criteria for pregnancy or lactation will be specified in the relevant DSA.

Where a DSA has an exclusion criterion for pregnancy, the following principles will apply:

1. In the event a pregnancy occurs whilst the participant is receiving the study intervention, the participant must discontinue the study intervention immediately.
2. If a participant becomes pregnant while on treatment with the study intervention, then the investigator should counsel the participant, discuss the risks of continuing with the pregnancy and the possible effects on the foetus.
3. The site investigator is responsible for reporting pregnancies that occur whilst the participant is receiving the study intervention within one working day of the site becoming aware of the event.

The participant is to be followed during the entire course of the pregnancy to report parental, foetal and neonatal outcomes.

4. Individual DSAs will specify if reporting is required for exposure to the study intervention during lactation and for the period of time, if any, that pregnancies require reporting following the last administration of the study intervention. Individual DSAs will also specify if pregnancies that occur in the partner of a participant require reporting.

11.4 Reporting

The principles of GCP require that both investigators and sponsors follow specific procedures when reporting adverse events in clinical trials. Reporting requirements are summarised below.

11.4.1 Event Reports

For all interventions within this platform, the period of observation for the collection of AEs will be from the time of randomisation until the completion of active follow-up at week 108. For SSIs, the period of observation is for the duration of the platform trial.

All AEs will be managed as per usual local clinical practice and the treatment specific guidance provided within each DSA. The investigator should follow-up the outcome of AEs until clinical recovery is complete and laboratory results have returned to baseline, or until progression of the event has been stabilised.

11.4.2 Expedited Reporting

The site investigator is responsible for reporting SUSARs, SSIs and USMs within one working day of the site becoming aware of the event. For DSAs with an exclusion criterion for pregnancy, pregnancies that occur whilst the participant is receiving the study intervention must also be reported within one working day of the site becoming aware of the event.

11.4.3 Non-Expedited Reporting

The following events require reporting in the eCRF, but do not require expedited reporting:

1. Any SAE that is not a SUSAR, but is considered by the site investigator to be attributable to a study intervention or study participation. These SARs are to be reported in the eCRF within 7 days of the site becoming aware of the event.
2. Trial outcome events that are SAEs are to be reported in the eCRF within 15 days of the site becoming aware of the event. These include cardiovascular events (cardiovascular deaths, hospitalised heart failure, myocardial infarction, stroke) and deaths from any cause. Due to the expected nature of these events in the trial population, trial outcome events are not considered to be SAEs for safety management or regulatory reporting purposes.
3. SAEs that lead to a change in treatment, but are <u>not</u> trial outcomes and <u>not</u> considered to be attributable to the study intervention or study participation. Treatment in this situation includes both the study intervention and standard of care therapy. These events are to be reported in the eCRF at the next study visit.
4. SAEs that are deemed by the site investigator to be an event of particular concern, but are <u>not</u> trial outcomes, are <u>not</u> considered to be attributable to the study intervention or study participation, and do <u>not</u> lead to a change in treatment. These events are to be reported in the eCRF at the next study visit.
5. Non-serious AEs that lead to the permanent discontinuation of the study intervention are to be reported on the eCRF at the next study visit.

11.4.4 Reporting of Adverse Events of Special Interest

Hyperkalaemia and AKI, as defined in Section 11.1.6 are to be reported in the eCRF at the next study visit. Shorter timelines for reporting may be required if a specific intervention significantly increases the risk of these events. In these cases, the shorter timelines for reporting will be detailed in the relevant DSA.

Any additional AESIs that are detailed in specific DSAs will be accompanied by reporting timelines in the DSA.

Should the AESI meet the definition of a SUSAR, SAR or trial outcome event, the shortest reporting timeline applies.

11.4.5 Exemptions to Reporting

The following events do not require reporting in the CAPTIVATE trial as they are not informative with respect to determining the safety of the study intervention or study participation. These events do not require reporting in the eCRF.

i. SAEs that are <u>not</u> trial outcomes, and which meet all of the following criteria:
    a. Are <u>not</u> considered to be attributable to the study intervention or study participation, and
    b. Do <u>not</u> lead to a change in standard of care or study intervention treatment, and
    c. Are <u>not</u> considered by the site investigator to be of particular concern.
ii. Non-serious AEs that do <u>not</u> lead to the permanent discontinuation of the study intervention.

11.4.6 Safety Reports

The GCC and/or RCC will submit annual safety reports in the form of a Development Safety Update Report (DSUR) to the relevant competent authorities where appropriate. Within some regions, these will only be required to be made available on request, where this is the case, the GCC or RCC will facilitate. Individual reports of events involving a study intervention supplied by an industry partner will be provided to the partner's safety team where applicable, as per contractual agreements.

12 STATISTICAL CONSIDERATIONS

12.1 Platform Trial Overview

CAPTIVATE is a multi-centre platform trial that tests multiple treatments simultaneously under a single overarching infrastructure. Treatments will be organized into domains, which consist of multiple mutually exclusive treatment options, referred to as interventions. Interventions will be evaluated on a background of standard of care therapy. Each domain will also include a control, usually standard of care alone.

Domains are described in a Domain-Specific Appendix (DSA) that contains information specific to the domain such as: eligibility criteria, features of the interventions and how they are delivered, and endpoints specific to the domain. Each domain may have adaptive features, such as dropping interventions for futility, declaring interventions superior, or response adaptive randomisation. These elements will be pre-specified in the DSA.

CAPTIVATE is designed as a perpetual platform trial and over time the domains enrolling within the platform may change. Domains may be added to the platform as they become scientifically appropriate and operationally feasible at the discretion of the POC. Domains will be removed when all investigational agents within the domain have met a pre-specified stopping criteria such as early futility/success or the maximum sample size and follow-up is reached.

Participants will be randomised among the interventions within each domain for which they are eligible (factorial randomisation). Participants eligible for multiple domains may receive multiple interventions at the same time. Each combination of interventions is referred to as a regimen. In addition to their assigned interventions, all participants will receive standard of care therapy as per local guideline recommendations.

A summary of the statistical methods that will be used in CAPTIVATE are presented in this section. Full details are located in the Statistical Analysis Appendix.

12.2 Statistical Analysis Overview

The primary analysis population for each domain is all randomised participants, regardless of adherence to the intervention, who were concurrently randomised and eligible for the domain. Data will be analysed according to intention-to-treat principles. Statistical analyses will be performed separately for each intervention.

The primary outcome is eGFR slope measured from randomisation through to week 108. eGFR at randomisation (baseline) is the average of two pre-randomisation values comprising the eGFR result from the randomisation visit and the eGFR result from the screening visit.

The treatment effect of each intervention will be estimated using a Bayesian linear mixed model. The treatment effect will be estimated during two periods, with an estimate of change in eGFR slope during the acute period (0-4 weeks) and the chronic period (5-108 weeks). For CAPTIVATE, the treatment effect of primary interest is the change in chronic eGFR slope.

A preliminary measure of efficacy, urine-albumin creatine ratio (uACR), will be measured at randomisation and 24 weeks. uACR at randomisation (baseline) is the average of 2 pre-randomisation values comprising the uACR result from the randomisation visit and the screening visit. uACR will be analysed as percentage

change from randomisation (%uACR), estimated using a linear model. The treatment benefit of each intervention on %uACR will be used to guide early adaptations within the domains.

12.3 Sample Size

Each intervention enrolled within the platform will have a maximum sample size specified within the associated DSA. The default sample size is 500 participants per intervention, which provides approximately 90% power to detect a 1.3 mL/min/1.73m2/year improvement in eGFR slope (additional details are provided in the Statistical Analysis Appendix). The total sample size of a domain is obtained by summing up the individual interventions; a domain with a single intervention and a control will have a maximum sample size of approximately 1,000 participants. The final sample size will depend on the simulations conducted prior to the DSA implantation and detailed in the Statistical Analysis Appendix.

When CAPTIVATE is initiated, each of the open domains is expected to consist of a single intervention and control. Therefore, the initial sample size of the platform is expected to be approximately 1,000 participants. The sample size will be increased as needed when additional domains are added to the platform.

Some domains may choose to share sample size between interventions. For example, if a domain consists of multiple doses of the same intervention, the DSA may specify a joint maximum sample size for those doses. This allows for more participants to be adaptively placed on the best intervention within that domain. This could occur if the domain uses response adaptive randomisation, or an intervention is dropped early due to futility.

12.4 Randomisation Principles

The randomisation scheme for each domain will be specified in the DSA. The default scheme is equal randomisation among the interventions and control (1:1: … :1). The algorithm used to achieve equal randomisation will be specified in the DSA. Randomisation will be stratified within each country. Should specific DSAs require additional stratification, this will be specified in the relevant DSA.

When the platform trial is initiated, it is anticipated that all domains will use fixed randomisation. However, response adaptive randomisation (RAR) may be specified with the DSA and the Statistical Analysis Appendix for future domains with two or more interventions. In accordance with best practice, RAR will be preceded by an initial data-collection/burn in period during which participants are allocated using fixed randomisation. The control arm will not be included in the RAR and will receive a fixed allocation, generally proportional to total number of interventions and control. This will lead to an upper limit on the allocation that an intervention can receive. For example, a hypothetical domain with three interventions would randomize 25% of patients to control, limiting the maximum randomisation to an intervention to at most 75%

Due to the length of the follow-up for the primary endpoint, RAR is expected to be guided by the supportive analysis of %uACR. Detailed simulations will be included in the DSA to show that the algorithm is properly implemented.

12.5 Analyses

[redacted]

[redacted]

12.5.1 Primary Analysis Population

[redacted]

[redacted]

[redacted]

12.5.2 Primary Analysis

[redacted]

[redacted]

[redacted]

[redacted]

[redacted]

12.5.4 Interim Analysis

[redacted]

[redacted]	[redacted]	[redacted]
[redacted]	[redacted]	[redacted]
[redacted]	[redacted]	[redacted]
[redacted]	[redacted]	[redacted]

[redacted]

[redacted]

[redacted]

[redacted]

12.5.5 Changes in the Standard of Care

[redacted]

[redacted]

13 ETHICAL CONSIDERATIONS

13.1 Ethics & Regulatory Compliance

The CAPTIVATE trial will be conducted according to the protocol, Good Clinical Practice (GCP) and in compliance with applicable laws and regulations. This protocol has been prepared in accordance with the SPIRIT statement: an international initiative that aims to improve the quality of clinical trial protocols by defining an evidence-based set of items to address in a protocol. www.spirit-statement.org/

This protocol has been developed by TGI and the CAPTIVATE POC. This document, and other trial documents where appropriate, including their future versions, will be reviewed and approved by the applicable Human Research Ethics Committee (HREC), Institutional Review Board (IRB) or similar with respect to scientific content and compliance with applicable regulations.

The trial will only be performed following the appropriate notification or approval where appropriate from the relevant regulatory body required locally. The trial will be performed in accordance with the appropriate national or regional guidelines set out by that regulatory body.

13.2 Confidentiality

The study will be conducted in accordance with applicable Privacy laws and regulations. The investigator and trial staff must ensure that participants' anonymity will be maintained, that their identities are protected from unauthorized parties and take measures to prevent accidental or premature destruction of study documents. All documents will be stored securely and only accessible by trial staff and authorised personnel.

On all trial-specific documents, other than the signed consent, the participant will be referred to by a unique trial-specific number and/or code in any database, not by name. Information linking the participant's medical data to database materials will be maintained in a secure location at the participating site. The key to code and recode participant identifiers will only be accessible to local site personnel. Any information that may identify a participant will be excluded from data presented in the public arena.

Identifiable data collected for the purpose of data linkage to administrative datasets will be stored in separate, encrypted databases with additional restrictions on access.

When archiving or processing data pertaining to trial staff and/or to the participants, the co-ordinating centre shall take all appropriate measures to safeguard and prevent access to this data by any unauthorized third party.

13.3 Insurance

The George Institute for Global Health certifies that it has a liability insurance policy in accordance with local laws and requirements.

13.4 Declaration of Interests

All trial staff will be required to declare and update all interests that might or might be seen to influence one or both of the conduct of the trial or the interpretation of results. A registry of interests will be maintained on the publicly accessible CAPTIVATE website. The Platform Chief Investigators will determine

whether a conflict of interest exists and the measures that are required to manage the conflict. If a Platform Chief Investigator declares a potential conflict of interest, the POC will determine measures for managing the conflict. Possible measures include disclosure in the relevant PISCF, requiring the individual to absent themselves from decision-making or reduce their role in the research, or requiring the individual to relinquish the interest.

14 REFERENCES

1. Roth GA, Mensah GA, Johnson CO, Addolorato G, Ammirati E, Baddour LM, et al. Global Burden of Cardiovascular Diseases and Risk Factors, 1990–2019: Update From the GBD 2019 Study. JACC. 2020;76(25):2982-3021.
2. Roth GA, Abate D, Abate KH, Abay SM, Abbafati C, Abbasi N, et al. Global, regional, and national age-sex-specific mortality for 282 causes of death in 195 countries and territories, 1980–2017: a systematic analysis for the Global Burden of Disease Study 2017. The Lancet. 2018;392(10159):1736-88.
3. Bikbov B, Purcell CA, Levey AS, Smith M, Abdoli A, Abebe M, et al. Global, regional, and national burden of chronic kidney disease, 1990–2017: a systematic analysis for the Global Burden of Disease Study 2017. The Lancet. 2020;395(10225):709-33.
4. Noble R, Taal MW. Epidemiology and causes of chronic kidney disease. Medicine. 2019;47(9):562-6.
5. Romagnani P, Remuzzi G, Glassock R, Levin A, Jager KJ, Tonelli M, et al. Chronic kidney disease. Nat Rev Dis Primers. 2017;3:17088.
6. Liyanage T, Ninomiya T, Jha V, Neal B, Patrice HM, Okpechi I, et al. Worldwide access to treatment for end-stage kidney disease: a systematic review. Lancet. 2015;385(9981):1975-82.
7. AIHW (Australian Institute of Health and Welfare). Disease expenditure in Australia 2018-19. AIHW, Australian Government; 2021.
8. Kidney Health Australia. National Strategic Action Plan for Kidney Disease. Available from kidney.org.au; 2019.
9. Foley RN, Parfrey PS, Sarnak MJ. Clinical epidemiology of cardiovascular disease in chronic renal disease. AJKD. 1998;32(5):S112-S9.
10. Savira F, Ademi Z, Wang BH, Kompa AR, Owen AJ, Liew D, et al. The Preventable Productivity Burden of Kidney Disease in Australia. J Am Soc Nephrol. 2021;32(4):938-49.
11. KDIGO. KDIGO 2012 Clinical Practice Guideline for the Evaluation and Management of Chronic Kidney Disease. Kidney International Supplements. 2013;3(1).
12. Wright JT, Jr., Bakris G, Greene T, Agodoa LY, Appel LJ, Charleston J, et al. Effect of blood pressure lowering and antihypertensive drug class on progression of hypertensive kidney disease: results from the AASK trial. Jama. 2002;288(19):2421-31.
13. Lewis EJ, Hunsicker LG, Clarke WR, Berl T, Pohl MA, Lewis JB, et al. Renoprotective effect of the angiotensin-receptor antagonist irbesartan in patients with nephropathy due to type 2 diabetes. N Engl J Med. 2001;345(12):851-60.
14. The GISEN Group. Randomised placebo-controlled trial of effect of ramipril on decline in glomerular filtration rate and risk of terminal renal failure in proteinuric, non-diabetic nephropathy. The Lancet. 1997;349(9069):1857-63.
15. Brenner BM, Cooper ME, de Zeeuw D, Keane WF, Mitch WE, Parving HH, et al. Effects of losartan on renal and cardiovascular outcomes in patients with type 2 diabetes and nephropathy. N Engl J Med. 2001;345(12):861-9.
16. Heart Outcomes Prevention Evaluation Study Investigators. Effects of ramipril on cardiovascular and microvascular outcomes in people with diabetes mellitus: results of the HOPE study and MICRO-HOPE substudy. Heart Outcomes Prevention Evaluation Study Investigators. Lancet. 2000;355(9200):253-9.
17. Xie X, Liu Y, Perkovic V, Li X, Ninomiya T, Hou W, et al. Renin-Angiotensin System Inhibitors and Kidney and Cardiovascular Outcomes in Patients With CKD: A Bayesian Network Meta-analysis of Randomized Clinical Trials. Am J Kidney Dis. 2016;67(5):728-41.

18. Oshima M, Neuen BL, Li J, Perkovic V, Charytan DM, de Zeeuw D, et al. Early Change in Albuminuria with Canagliflozin Predicts Kidney and Cardiovascular Outcomes: An Analysis from the CREDENCE Trial. JASN. 2020;31(12):2925.
19. Levey AS, Inker LA, Matsushita K, Greene T, Willis K, Lewis E, et al. GFR decline as an end point for clinical trials in CKD: a scientific workshop sponsored by the National Kidney Foundation and the US Food and Drug Administration. Am J Kidney Dis. 2014;64(6):821-35.
20. Tangri N, Stevens LA, Griffith J, Tighiouart H, Djurdjev O, Naimark D, et al. A predictive model for progression of chronic kidney disease to kidney failure. Jama. 2011;305(15):1553-9.
21. Levey AS, de Jong PE, Coresh J, El Nahas M, Astor BC, Matsushita K, et al. The definition, classification, and prognosis of chronic kidney disease: a KDIGO Controversies Conference report. Kidney International. 2011;80(1):17-28.
22. Heerspink HJL, Greene T, Tighiouart H, Gansevoort RT, Coresh J, Simon AL, et al. Change in albuminuria as a surrogate endpoint for progression of kidney disease: a meta-analysis of treatment effects in randomised clinical trials. The lancet Diabetes & endocrinology. 2019;7(2):128-39.
23. Inker LA, Heerspink HJL, Tighiouart H, Levey AS, Coresh J, Gansevoort RT, et al. GFR Slope as a Surrogate End Point for Kidney Disease Progression in Clinical Trials: A Meta-Analysis of Treatment Effects of Randomized Controlled Trials. J Am Soc Nephrol. 2019;30(9):1735-45.
24. Greene T, Ying J, Vonesh EF, Tighiouart H, Levey AS, Coresh J, et al. Performance of GFR Slope as a Surrogate End Point for Kidney Disease Progression in Clinical Trials: A Statistical Simulation. J Am Soc Nephrol. 2019;30(9):1756-69.
25. González AM, Gutman T, Lopez-Vargas P, Anumudu S, Arce CM, Craig JC, et al. Patient and Caregiver Priorities for Outcomes in CKD: A Multinational Nominal Group Technique Study. AJKD. 2020;76(5):679-89.
26. Levey AS, Gansevoort RT, Coresh J, Inker LA, Heerspink HL, Grams ME, et al. Change in Albuminuria and GFR as End Points for Clinical Trials in Early Stages of CKD: A Scientific Workshop Sponsored by the National Kidney Foundation in Collaboration With the US Food and Drug Administration and European Medicines Agency. Am J Kidney Dis. 2020;75(1):84-104.
27. Travere Therapeutics. Travere Therapeutics Announces FDA Accelerated Approval of FILSPARI™ (sparsentan), the First and Only Non-immunosuppressive Therapy for the Reduction of Proteinuria in IgA Nephropathy San Diego: Travere Therapeutics; 2023 [updated 17 February 2023. Available from: https://ir.travere.com/news-releases/news-release-details/travere-therapeutics-announces-fda-accelerated-approval.
28. U.S. Food & Drug Administration. FDA approves first drug to decrease urine protein in IgA nephropathy, a rare kidney disease US: US FDA; 2021 [updated 17 December 2021. Available from: https://www.fda.gov/drugs/fda-approves-first-drug-decrease-urine-protein-iga-nephropathy-rare-kidney-disease.
29. Heerspink HJL, Radhakrishnan J, Alpers CE, Barratt J, Bieler S, Diva U, et al. Sparsentan in patients with IgA nephropathy: a prespecified interim analysis from a randomised, double-blind, active-controlled clinical trial. The Lancet. 2023;401(10388):1584-94.
30. Barratt J, Lafayette R, Kristensen J, Stone A, Cattran D, Floege J, et al. Results from part A of the multi-center, double-blind, randomized, placebo-controlled NefIgArd trial, which evaluated targeted-release formulation of budesonide for the treatment of primary immunoglobulin A nephropathy. Kidney Int. 2023;103(2):391-402.
31. The George Institute for Global Health. Global Kidney Patients Trials Network (GKPTN) Sydney2021 [Available from: https://www.georgeinstitute.org/projects/global-kidney-patients-trials-network-gkptn.

32. ISN. New Global Collaboration to Accelerate Treatments for Kidney Disease 2021 [Available from: https://www.theisn.org/blog/2021/10/18/new-global-collaboration-to-accelerate-treatments-for-kidney-disease/.
33. Kumar V, Yadav AK, Sethi J, Ghosh A, Sahay M, Prasad N, et al. The Indian Chronic Kidney Disease (ICKD) study: baseline characteristics. Clin Kidney J. 2022;15(1):60-9.
34. Perkovic V, Craig JC, Chailimpamontree W, Fox CS, Garcia-Garcia G, Benghanem Gharbi M, et al. Action plan for optimizing the design of clinical trials in chronic kidney disease. Kidney Int Suppl (2011). 2017;7(2):138-44.
35. Levin A, Tonelli M, Bonventre J, Coresh J, Donner JA, Fogo AB, et al. Global kidney health 2017 and beyond: a roadmap for closing gaps in care, research, and policy. Lancet. 2017;390(10105):1888-917.
36. Woodcock J, LaVange LM. Master Protocols to Study Multiple Therapies, Multiple Diseases, or Both. N Engl J Med. 2017;377(1):62-70.
37. Kotwal S, Perkovic V, Heerspink HJL. Platform Clinical Trials Within Nephrology-Interpreting the Evidence. AJKD. 2022;80(1):143-6.
38. Berry SM, Connor JT, Lewis RJ. The platform trial: an efficient strategy for evaluating multiple treatments. JAMA. 2015;313(16):1619-20.
39. Ware JEJ, Richardson MM, Meyer KB, Gandek B. Improving CKD-Specific Patient-Reported Measures of Health-Related Quality of Life. Journal of the American Society of Nephrology. 2019;30(4):664-77.

15 PROTOCOL AMENDMENTS

Any modifications to the protocol which may impact on the conduct of the trial, potential benefit of the participant or may affect participant safety, including changes of trial objectives, trial design, patient population, sample sizes, trial procedures, or significant administrative aspects will require a formal amendment to the protocol. Such amendment will be agreed upon by the POC, and approved by the relevant ethics committees prior to implementation, and notified to regulatory authorities in accordance with local regulations.

The full history of modifications to the CAPTIVATE core protocol is described in the table below;

Version & Date	Changes from Prior Version
**v1.1, 20Jul2023**	N/A
**v2.0, 16Nov2023**	Minor editorial changes and typographic corrections made throughout the protocol for consistency and clarity.
	Section 3.1: Updated platform secondary outcomes and platform exclusion criteria as per amendments to Section 7.3.2 and Section 7.5.2
	Section 3.2: Updated Sponsor address. Added NHMRC grant identification number and ClinicalTrials.gov identifier.
	Section 3.3.4: Clarified that the Core Statistical Design and Simulation Report is the Statistical Analysis Appendix
	Section 6.2: Updated secondary objectives to align with secondary outcomes in Section 7.5.2
	Section 7.3.2: Added exclusion criterion to exclude patients who are currently receiving maintenance dialysis. This is implied by the eGFR inclusion criterion.
	Section 7.5: Added flexibility should a domain be unable to implement all core outcomes
	Section 7.5.2: Added additional time to event secondary outcomes for kidney and cardiovascular events. Amended the outcome for number of cardiovascular events to proportion of participants experiencing one or more cardiovascular events. Clarified that uACR at randomisation is the average of uACR results at the screening and randomisation visits.
	Section 8.2: Added flexibility should a domain be unable to implement the full core schedule of assessments. Clarified that urine samples for urinalysis should be collected from the first void of the day where possible.
	Section 9.1: Clarified that trial data will be stored on TGI servers.
	Section 9.2.1 and Section 10.1.3: Increased retention period for trial-related documentation to 25 years to align with EU clinical trial regulations.
	Section 11: Added details about the different levels of safety monitoring.
	Section 11.4.4: Clarified that if a AESI meets the definition of a SUSAR, SAR or trial outcome event, the shortest reporting timeline applies.

16 APPENDIX

16.1 Outcome Definitions for Cardiovascular Events

16.1.1 Cardiovascular (CV) death (1)

CV death to acute myocardial infarction (MI)
Death occurring within 30 days of an acute MI (defined in Section 16.1.3) by any CV mechanism as a direct consequence of the MI. This includes death resulting from an emergency procedure (e.g. percutaneous coronary intervention or coronary artery bypass graft surgery) to treat an MI.

Sudden cardiac death
Death that occurs unexpectedly and not within 30 days of an acute MI.

The following scenarios are included:

- Witnessed death with no new or progressive symptoms.
- Witnessed death within 60 minutes of new cardiac symptoms not related to an acute MI.
- Witnessed death with an identifiable causal cardiac arrhythmia in a previously clinically stable individual.
- Death within 7 days of successful cardiac resuscitation without an identifiable aetiology (cardiac or otherwise).
- Unwitnessed death within 24 hours of being seen alive and clinically stable with no symptoms.

CV death due to heart failure (HF)
Death associated with progressive HF with clinically worsening symptoms and/or signs, and/or increased therapeutic requirements including medication and mechanical circulatory support.

CV death due to stroke
Death following a stroke (defined in Section 16.1.4) that is a direct consequence or a complication of the stroke.

CV death due to a cardiovascular procedure
Death caused by the immediate complication(s) of a CV procedure.

CV death due to other cardiovascular cause
Death associated with other specific known CV causes (e.g. CV haemorrhage, pulmonary embolism or peripheral arterial disease) that are not included in the above categories.

16.1.2 Hospitalised heart failure (1, 2)

An unplanned inpatient hospital admission for a primary diagnosis of HF. All the following criteria must be fulfilled:

- The length of stay is greater than 24 hours or, where the admission and discharge times are unavailable, is over at least 2 calendar days.
- The individual reports clinical symptoms typical of HF (e.g. dyspnoea, reduced exercise tolerance, fatigue).
- The individual exhibits clinical signs typical of HF (e.g. peripheral oedema, ascites, pulmonary crackles/crepitations, raised jugular venous pressure, rapid weight gain related to fluid overload).

- The diagnostic test results are consistent with HF (e.g. elevated natriuretic peptides, radiological features of pulmonary congestion, echocardiographic or invasive evidence of raised ventricular filling pressures or low cardiac output).
- The individual receives treatment for HF (e.g. oral and/or intravenous diuretic therapy, vasoactive therapy, mechanical circulatory support, mechanical fluid removal).

16.1.3 Myocardial infarction (MI) (1, 3)

The presence of myocardial necrosis proven by elevated cardiac biomarkers with clinical findings consistent with acute myocardial injury based on presentation, electrocardiographic changes (ECG), and/or non-invasive or invasive myocardial or coronary artery imaging. The clinical classification of MI are outlined below.

Type 1 MI
Elevated cardiac troponin value above the 99th percentile of the upper limit of normal plus at least 1 of the following clinical criteria:

- Symptoms of acute MI.
- New ECG abnormalities including ischaemic changes or pathological Q waves.
- Imaging (including echocardiographic) evidence of new ischaemic pathology including loss of viable myocardium or regional wall motion abnormality.
- Presence of coronary thrombus on angiography or autopsy.

Type 2 MI
Ischaemic myocardial injury due to insufficient myocardial oxygen supply in the absence of acute atherothrombotic plaque disruption with an elevated cardiac troponin value above the 99th percentile of the upper limit of normal plus at least 1 of the following criteria:

- Symptoms of acute MI.
- New ECG abnormalities including ischaemic changes or pathological Q waves.
- Imaging (including echocardiographic) evidence of new ischaemic pathology including loss of viable myocardium or regional wall motion abnormality.

Type 3 MI
CV death with preceding symptoms and ECG changes consistent with acute MI occurring before a rise in cardiac biomarkers can be detected, blood samples can be collected or MI is confirmed on autopsy.

Type 4a MI
Percutaneous coronary intervention (PCI) related MI within 48 hours of the index procedure with a rise in cardiac troponin by > 5 times the 99th percentile of the upper limit of normal and, in individuals with elevated but stable/falling pre-procedure values, a post-procedure value rise of > 20%. In addition, at least 1 of the following criteria is required:

- New ECG abnormalities including ischaemic changes or pathological Q waves.
- Imaging (including echocardiographic) evidence of new ischaemic pathology including loss of viable myocardium or regional wall motion abnormality.
- Angiographic evidence of a procedural flow-limiting complication.

Type 4b MI
Stent or scaffold thrombosis associated with PCI confirmed on angiography or autopsy, applying the same criteria used for Type 1 MI.

Type 4c MI
In-stent restenosis or restenosis following coronary balloon angioplasty in the infarct territory, applying the same criteria used for Type 1 MI.

Type 5 MI
Coronary artery bypass graft related MI occurring within 48 hours of the index procedure with a rise in cardiac troponin by > 10 times the 99th percentile of the upper limit of normal and, in individuals with elevated but stable/falling pre-procedure values, a post-procedure value rise of > 20%. In addition, at least 1 of the following criteria is required:

- New pathological Q waves on ECG.
- Imaging (including echocardiographic) evidence of new ischaemic pathology including loss of viable myocardium or regional wall motion abnormality.
- Angiographic evidence of a new graft occlusion or new native coronary artery occlusion.

16.1.4 Stroke (1, 4)

Ischaemic Stroke
An acute episode of cerebral, spinal or retinal dysfunction as a result of central nervous system tissue infarction with at least 1 of the following criteria:

- Pathological or radiological evidence of neurological ischaemic injury in a defined vascular distribution.
- Persistent symptoms for ≥ 24 hours without other identifiable aetiologies.

Haemorrhagic Stroke
An acute episode of cerebral or spinal dysfunction as a result of intraparenchymal, intraventricular or subarachnoid haemorrhage.

Undetermined Stroke
An acute episode of neurological dysfunction presumed to be caused by brain, spinal cord or retinal vascular injury due to haemorrhage or infarction with insufficient information to allow categorisation.

1 Hicks KA, Mahaffey KW, Mehran R, Nissen SE, Wiviott SD, Dunn B, Solomon SD, Marler JR, Teerlink JR, Farb A, Morrow DA, Targum SL, Sila CA, Hai MTT, Jaff MR, Joffe HV, Cutlip DE, Desai AS, Lewis EF, Gibson CM, Landray MJ, Lincoff AM, White CJ, Brooks SS, Rosenfield K, Domanski MJ, Lansky AJ, McMurray JJV, Tcheng JE, Steinhubl SR, Burton P, Mauri L, O'Connor CM, Pfeffer MA, Hung HMJ, Stockbridge NL, Chaitman BR, Temple RJ; Standardized Data Collection for Cardiovascular Trials Initiative (SCTI). 2017 Cardiovascular and Stroke Endpoint Definitions for Clinical Trials. Circulation. 2018 Feb 27;137(9):961-972. doi: 10.1161/CIRCULATIONAHA.117.033502. PMID: 29483172.

2 Abraham WT, Psotka MA, Fiuzat M, Filippatos G, Lindenfeld J, Mehran R, Ambardekar AV, Carson PE, Jacob R, Januzzi JL Jr, Konstam MA, Krucoff MW, Lewis EF, Piccini JP, Solomon SD, Stockbridge N, Teerlink JR, Unger EF, Zeitler EP, Anker SD, O'Connor CM. Standardized Definitions for Evaluation of Heart Failure Therapies: Scientific Expert Panel From the Heart Failure Collaboratory and Academic Research Consortium. JACC Heart Fail. 2020 Dec;8(12):961-972. doi: 10.1016/j.jchf.2020.10.002. Epub 2020 Nov 13. PMID: 33199251.

3 Thygesen K, Alpert JS, Jaffe AS, Chaitman BR, Bax JJ, Morrow DA, White HD; Executive Group on behalf of the Joint European Society of Cardiology (ESC)/American College of Cardiology (ACC)/American Heart Association (AHA)/World Heart Federation (WHF) Task Force for the Universal Definition of Myocardial Infarction. Fourth Universal Definition of Myocardial Infarction (2018). Circulation. 2018 Nov 13;138(20):e618-e651. doi: 10.1161/CIR.0000000000000617. Erratum in: Circulation. 2018 Nov 13;138(20):e652. PMID: 30571511.

4 Sacco RL, Kasner SE, Broderick JP, Caplan LR, Connors JJ, Culebras A, Elkind MS, George MG, Hamdan AD, Higashida RT, Hoh BL, Janis LS, Kase CS, Kleindorfer DO, Lee JM, Moseley ME, Peterson ED, Turan TN, Valderrama AL, Vinters HV; American Heart Association Stroke Council, Council on Cardiovascular Surgery and Anesthesia; Council on Cardiovascular Radiology and Intervention; Council on Cardiovascular and Stroke Nursing; Council on Epidemiology and Prevention; Council on Peripheral Vascular Disease; Council on Nutrition, Physical Activity and Metabolism. An updated definition of stroke for the 21st century: a statement for healthcare professionals from the American Heart Association/American Stroke Association. Stroke. 2013 Jul;44(7):2064-89. doi: 10.1161/STR.0b013e318296aeca. Epub 2013 May 7. Erratum in: Stroke. 2019 Aug;50(8):e239. PMID: 23652265.

16.2 Quality of Life Impact Survey for Kidney Disease (QDIS-CKD)

Quality of Life Impact Survey for Kidney Disease

1. In the past 4 weeks, how much did your kidney disease limit your everyday activities or your quality of life?

Not at all	A little	Some	A lot	Extremely
☐ 1	☐ 2	☐ 3	☐ 4	☐ 5

2. In the past 4 weeks, how often…

	Never	Rarely	Sometimes	Often	Very often
a. Did kidney disease limit your usual physical activities?	☐ 1	☐ 2	☐ 3	☐ 4	☐ 5
b. Did you have difficulty doing work or other daily activities because of kidney disease?	☐ 1	☐ 2	☐ 3	☐ 4	☐ 5
c. Did kidney disease make you worn out or too tired to work or do daily activities?	☐ 1	☐ 2	☐ 3	☐ 4	☐ 5
d. Did kidney disease limit your usual social activities with family, friends, or others close to you?	☐ 1	☐ 2	☐ 3	☐ 4	☐ 5
e. Did you feel frustrated or fed up because of kidney disease?	☐ 1	☐ 2	☐ 3	☐ 4	☐ 5
f. Did kidney disease make you worry about your health or future health problems?	☐ 1	☐ 2	☐ 3	☐ 4	☐ 5

Thank you for answering these questions!

7-item Disease-specific Quality of Life Impact Scale (QDIS®-7) for Kidney Disease, United States (English)
Copyright © 2010-2019 John Ware Research Group, Inc. All rights reserved. QDIS® is a registered trademark of John Ware Research Group, Inc.
