# Supplementary material for: The Global Kidney Patient Trials Network and the CAPTIVATE Platform Clinical Trial Design: A Trial Protocol
Source: JAMA Netw Open. 2024 Dec 11;7(12):e2449998. doi: 10.1001/jamanetworkopen.2024.49998 (PMC11635535; doi:10.1001/jamanetworkopen.2024.49998)
Supplement: Supplement 3. — Nonauthor Collaborators [file jamanetwopen-e2449998-s003.pdf]

Supplemental Online Content: Nonauthor Collaborators
*First name, last name, and suffix (if applicable) are required and will appear in PubMed.

*Group Name(s): The GKPTN and CAPTIVATE Investigators							
***First Name and Middle Initial(s)**	***Last Name**	***Suffix (eg, Jr, III)**	Academic Degrees	Institution	Location (city, state/province, country)	Role or Contribution, eg, chair, principal investigator	Group (if more than 1 Group listed in the byline) and/or Subgroup (eg, Steering Committee)
Andres	Alvarisqueta		MD	Centro de Investigaciones Medicas Mar del Plata	Argentina	Principal Investigator	GKPTN
Julio	Bittar			CERECA	Argentina	Principal Investigator	GKPTN
Natalia	Cluigt			Instituto de Investigaciones Clinicas Mar del Plata	Argentina	Principal Investigator	GKPTN
Judith	Gaite			Clinica de Nefrologia, Urologia y Enfermedades	Argentina	Principal Investigator	GKPTN
Luis	Gaite			Clinica de Nefrologia, Urologia y Enfermedades	Argentina	Principal Investigator	GKPTN
Silvia Marcela	Maurich			Inst de Cardiología de Corrientes Juana Francisca Cabral	Argentina	Principal Investigator	GKPTN
Pablo	Ramallo			Centro Modelo de Cardiologia	Argentina	Principal Investigator	GKPTN
Alejandra	Quevedo			Investigacion Clinica Aplicada SRL	Argentina	Principal Investigator	GKPTN
Carlos	Arias			Centro de Nutrición y Diabetes (CENUDIAB)	Argentina	Principal Investigator	GKPTN
Jorge Hector	Resk			Well Medica (previously known as CICIC- Centro de Investigaciones Clínicas Instituto del Corazón)	Argentina	Principal Investigator	GKPTN
Mariana	Brusa			Well Medica (previously known as CICIC- Centro de Investigaciones Clínicas Instituto del Corazón)	Argentina	Principal Investigator	GKPTN
Paula Andrea	Marioli			Renalida	Argentina	Principal Investigator	GKPTN
Lawrence	McMahon			Eastern Health	Australia	Principal Investigator	GKPTN
Sridevi	Govindarajulu			Toowoomba Hospital	Australia	Principal Investigator	GKPTN

Supplemental Online Content: Nonauthor Collaborators
*First name, last name, and suffix (if applicable) are required and will appear in PubMed.

*First Name and Middle Initial(s)	*Last Name	*Suffix (eg, Jr, III)	Academic Degrees	Institution	Location (city, state/province, country)	Role or Contribution, eg, chair, principal investigator	Group (if more than 1 Group listed in the byline) and/or Subgroup (eg, Steering Committee)
Nicholas	Gray			Sunshine Coast University Hospital	Australia	Principal Investigator;	GKPTN
Simon	Roger			Renal Research	Australia	Principal Investigator	GKPTN
Adam	Flavell			Western Health	Australia	Principal Investigator	GKPTN
Suda	Swaminathan			Fiona Stanley Hospital	Australia	Principal Investigator	GKPTN
Nigel	Toussaint			The Royal Melbourne Hospital	Australia	Principal Investigator	GKPTN
Jessica	Stranks			Lyell McEwin Hospital	Australia	Principal Investigator	GKPTN
Peak Mann	Mah			Lyell McEwin Hospital	Australia	Principal Investigator	GKPTN
Parind	Vora			Lyell McEwin Hospital	Australia	Principal Investigator	GKPTN
Serge	Cournoyer			CISSS de la Montérégie-Centre	Canada	Principal Investigator	GKPTN
Marie-France	Langlois			Sherbrooke University	Canada	Principal Investigator	GKPTN
Matthew	Weir			London Health Sciences Centre	Canada	Principal Investigator	GKPTN
Fan Fan	Hou			Nanfang Hospital, Southern Medical University	China	Principal Investigator;	GKPTN
Hong	Zhang			Peking University First Hospital	China	Principal Investigator;	GKPTN
Zhihong	Liu			Affiliated Jinling Hospital, Medical School of Nanjing University	China	National Lead	GKPTN
Wang	Caili			The First affiliated hospital of Baotao Medical College, Inner Mongolia University of Science and Technology	China	Principal Investigator	GKPTN
Luo	Qun			Ningbo Huamei Hospital, University of Chinese Academy of Sciences	China	Principal Investigator	GKPTN
Kong	Yaozhong			The First People's Hospital of Foshan	China	Principal Investigator	GKPTN
Fu	Ping			West China Hospital of Sichuan University	China	Principal Investigator	GKPTN
Xiong	Zuying			Peiking University Shenzhen Hospital	China	Principal Investigator	GKPTN

Supplemental Online Content: Nonauthor Collaborators
*First name, last name, and suffix (if applicable) are required and will appear in PubMed.

*First Name and Middle Initial(s)	*Last Name	*Suffix (eg, Jr, III)	Academic Degrees	Institution	Location (city, state/province, country)	Role or Contribution, eg, chair, principal investigator	Group (if more than 1 Group listed in the byline) and/or Subgroup (eg, Steering Committee)
Lu	Wanhong			First Affiliated Hospital of Xi’an Jiaotong University	China	Principal Investigator	GKPTN
Li	Guisen			Sichuan Provincial People's Hospital	China	Principal Investigator	GKPTN
Menghua	Chen			General Hospital of Ningxia Medical University	China	Principal Investigator	GKPTN
Li	Peng			Yantai Yuhuangding hospital	China	Principal Investigator	GKPTN
Yiwen	Liu			Zhejiang Provincial People’s Hospital	China	Principal Investigator	GKPTN
Cheng	Hong			Beijing Anzhen Hospital affiliated to Beijing Capital Medical University	China	Principal Investigator	GKPTN
Jianqin	Wang			The Second Hospital of Lanzhou University	China	Principal Investigator	GKPTN
Dong	Junwu			Wuhan Fourth Hospital (Wuhan Puai Hospital, Wuhan Orthopedic Hospital)	China	Principal Investigator	GKPTN
Ma	Zhigang			Gansu Provincial People's Hospital	China	Principal Investigator	GKPTN
Yan	Rui			Affiliated Hospital of Guizhou Medical University	China	Principal Investigator	GKPTN
Shi	Yongjun			Huizhou Central People's Hospital	China	Principal Investigator	GKPTN
Zhang	Chun			Union Hospital, Tongji Medical College, Huazhong University of Science and Technology	China	Principal Investigator	GKPTN
Lv	Xueai			Taian City Central Hospital	China	Principal Investigator	GKPTN
Yu	Xiaoyong			Shaanxi Provincial Hospital of traditional Chinese Medicine	China	Principal Investigator	GKPTN
Bian	Xueyan			Ningbo First Hospital	China	Principal Investigator	GKPTN

Supplemental Online Content: Nonauthor Collaborators
*First name, last name, and suffix (if applicable) are required and will appear in PubMed.

***First Name and Middle Initial(s)**	***Last Name**	***Suffix (eg, Jr, III)**	Academic Degrees	Institution	Location (city, state/province, country)	Role or Contribution, eg, chair, principal investigator	Group (if more than 1 Group listed in the byline) and/or Subgroup (eg, Steering Committee)
Yihua	Bai			The Second Affiliated Hospital of Kunming Medical University	China	Principal Investigator	GKPTN
Maura	Ravera			Ospedale Policlinico San MartiN –IRCCS	Italy	Principal Investigator	GKPTN
Antonio	Pisani			Università degli Studi di Napoli Federico II	Italy	Principal Investigator	GKPTN
Mariacristina	Gregorini			Arcispedale Santa Maria Nuova Azienda USL-IRCCS	Italy	Principal Investigator	GKPTN
Ciro	Esposito			ICS Salvatore Maugeri SpA SB	Italy	Principal Investigator	GKPTN
Filippo	Aucella			Ospedale Casa Sollievo della Sofferenza - IRCCS	Italy	Principal Investigator	GKPTN
Luca	De Nicola			Ospedale S. Maria degli Incurabili Università degli studi della Campania Luigi Vanvitelli	Italy	Principal Investigator; [illegible]	GKPTN
Felice	Nappi			Ospedaliero S. Maria della Pietà	Italy	Principal Investigator	GKPTN
Cataldo	Abaterusso			Ospedale S. Giacomo Apostolo di Castelfranco Veneto Azienda ULSS n. 2 Marca trevigiana	Italy	Principal Investigator	GKPTN
Loreto	Gesualdo			Azienda Universitaria Ospedaliera Consorziale - Policlinico Bari	Italy	Principal Investigator	GKPTN
Michele	Andreucci			Università degli Studi Magna Graecia di Catanzaro Azienda Ospedaliero - Universitaria "Mater Domini"	Italy	Principal Investigator	GKPTN
Mariadelina	Simeoni			Università degli studi della Campania Luigi Vanvitelli	Italy	Principal Investigator	GKPTN

Supplemental Online Content: Nonauthor Collaborators
*First name, last name, and suffix (if applicable) are required and will appear in PubMed.

***First Name and Middle Initial(s)**	***Last Name**	***Suffix (eg, Jr, III)**	Academic Degrees	Institution	Location (city, state/province, country)	Role or Contribution, eg, chair, principal investigator	Group (if more than 1 Group listed in the byline) and/or Subgroup (eg, Steering Committee)
Seiji	Itano			Kawasaki Medical School Hospital	Japan	Principal Investigator	GKPTN
Naoki	Kashihara			Kawasaki Medical School Hospital	Japan	Principal Investigator	GKPTN
Jun	Wada			Okayama University	Japan	Principal Investigator	GKPTN
Yuka	Sugawara			The University of Tokyo Hospital	Japan	Principal Investigator;	GKPTN
Masaomi	Nangaku			The University of Tokyo Hospital	Japan	Principal Investigator	GKPTN
Motoji	Naka			Asama General Hospital	Japan	Principal Investigator	GKPTN
Masahiko	Takai			Takai Internal Medicine Clinic	Japan	Principal Investigator	GKPTN
Shin	Goto			Niigata University	Japan	Principal Investigator	GKPTN
Ichei	Narita			Niigata University	Japan	Principal Investigator	GKPTN
Masafumi	Fukagawa			Tokai University	Japan	Principal Investigator	GKPTN
Takashi	Yokoo			The Jikei University Hospital	Japan	Principal Investigator	GKPTN
Shinya	Kaname			Kyorin University	Japan	Principal Investigator	GKPTN
Abe	Masanori			Nihon University Itabashi Hospital	Japan	Principal Investigator	GKPTN
Yusuke	Suzuki			Juntendo University Hospital	Japan	Principal Investigator	GKPTN
María Jose	Soler Romeo			Hospital Vall d'Hebron	Spain	Principal Investigator	GKPTN
Emma	Pardo			Hospital Vall d'Hebron	Spain	Study Coordinator	GKPTN

Supplemental Online Content: Nonauthor Collaborators
*First name, last name, and suffix (if applicable) are required and will appear in PubMed.

*First Name and Middle Initial(s)	*Last Name	*Suffix (eg, Jr, III)	Academic Degrees	Institution	Location (city, state/province, country)	Role or Contribution, eg, chair, principal investigator	Group (if more than 1 Group listed in the byline) and/or Subgroup (eg, Steering Committee)
Alfonso	Soto Gonzalez			Hospital de A Coruña	Spain	Principal Investigator	GKPTN
Josep Maria	Cruzado			Hospital Bellvitge	Spain	Principal Investigator	GKPTN
Jose Luis	Górriz			Hospital Clinico Universitario de Valencia	Spain	Principal Investigator;	GKPTN
Secundino	Cigarran			Hospital da Costa (Burela) / Hospital Ribera Polusa (new location)	Spain	Principal Investigator	GKPTN
Fernando	Cereto Castro			Hospital Quiron Barcelona	Spain	Principal Investigator	GKPTN
Jonay	Pantoja Perez			Hospital Universitario Dr. Peset	Spain	Principal Investigator	GKPTN
Cristobal	Morales Portillo			Hospital Universitario Virgen Macarena	Spain	Principal Investigator	GKPTN
Francisco Jose	Tinahones Maduen			Hospital Virgen de la Victoria	Spain	Principal Investigator	GKPTN
Maria	Marques			Hospital Puerta de Hierro	Spain	Principal Investigator	GKPTN
Roberto	Pecoits-Filho			Arbor Research Collaborative for Health	USA	National Lead	GKPTN
Sergio	Rovner			Academy of Diabetes Thyroid and Endocrine, PA	USA	Principal Investigator	GKPTN
Ahmed	Arif			Ahmed Arif Medical Research Center	USA	Principal Investigator	GKPTN
Pablo	Pergola			Clinical Advancement Center, PLLC	USA	Principal Investigator	GKPTN
Tuan-Huy	Tran			Omega Clinical Research	USA	Principal Investigator	GKPTN
Manuel	Montero			Eastern Nephrology Associates - New Bern	USA	Principal Investigator	GKPTN

*First name, last name, and suffix (if applicable) are required and will appear in PubMed.

***First Name and Middle Initial(s)**	***Last Name**	***Suffix (eg, Jr, III)**	Academic Degrees	Institution	Location (city, state/province, country)	Role or Contribution, eg, chair, principal investigator	Group (if more than 1 Group listed in the byline) and/or Subgroup (eg, Steering Committee)
Jamal	Hammoud			Elite Clinical Research	USA	Principal Investigator	GKPTN
Michael	Shanik			Endocrine Associates of Long Island, PC	USA	Principal Investigator	GKPTN
Pedro Andres	Velasquez-Mieyer			LifeDOC Research PLLC	USA	Principal Investigator	GKPTN
Katherine Jean	Lucas			Lucas Research, Inc.	USA	Principal Investigator	GKPTN
James	Franklin			Medication Management LLC - Greensboro	USA	Principal Investigator	GKPTN
Arthur	Green			Medication Management LLC - Greensboro	USA	Principal Investigator	GKPTN
Andrew	Drabick			Medication Management, LLC - Raleigh Location	USA	Principal Investigator	GKPTN
Joseph	Alello			Mountain Kidney & Hypertension Associates	USA	Principal Investigator	GKPTN
Robert	Busch			Albany Medical College	USA	Principal Investigator	GKPTN
Nina	Patel			Rancho Research Institute	USA	Principal Investigator	GKPTN
Sanjay	Vora			SV Research LLC	USA	Principal Investigator	GKPTN
Osvaldo A.	Brusco			The Office of O. Alejandro Brusco	USA	Principal Investigator	GKPTN
Jose	Gomez-Cortez			Universal Clinical Research	USA	Principal Investigator	GKPTN
Csaba	Kovesdy			Clinical Trials Network of Tennessee	USA	Principal Investigator	GKPTN
Ian	de Boer			University of Washington	USA	Principal Investigator	GKPTN

Supplemental Online Content: Nonauthor Collaborators
*First name, last name, and suffix (if applicable) are required and will appear in PubMed.

***First Name and Middle Initial(s)**	***Last Name**	***Suffix (eg, Jr, III)**	Academic Degrees	Institution	Location (city, state/province, country)	Role or Contribution, eg, chair, principal investigator	Group (if more than 1 Group listed in the byline) and/or Subgroup (eg, Steering Committee)
Radica	Alicic			Providence Sacred Heart	USA	Principal Investigator	GKPTN
Eric	Kirk			Eastern Nephrology Associates - Wilmington	USA	Principal Investigator	GKPTN
Nauman	Shahid			Eastern Nephrology Associates - Greenville	USA	Principal Investigator	GKPTN
Anand	Reddy			Permian Research Foundation	USA	Principal Investigator	GKPTN
Pedro	Hernandez			Y & L Advance Health Care Inc. D/B /A Elite Clinical Research	USA	Principal Investigator	GKPTN
Ronald	Mayfield			Tribe Clinical Research d/b/a Mountain View Clinical Research	USA	Principal Investigator	GKPTN
Linda	Schneider			TPMG Clinical Research	USA	Principal Investigator	GKPTN
Brian	Layden			Jesse Brown VA Medical Center	USA	Principal Investigator	GKPTN
Gerard	Bueso			Endocrine Associates	USA	Principal Investigator	GKPTN
Margaret	Yu			Stanford University	USA	Principal Investigator	GKPTN
Vinod	Malhotra			HB Clinical Trials, Inc	USA	Principal Investigator	GKPTN
Billy	Hour			Amicis Research Center	USA	Principal Investigator	GKPTN
Kianoosh	Kaveh			Volunteer Medical Research	USA	Principal Investigator	GKPTN
Visal	Numrungroad			Suncoast Medical Research, Inc.	USA	Principal Investigator	GKPTN
Reginald	Gohh			Lifespan - Rhode Island Hospital	USA	Principal Investigator	GKPTN

Supplemental Online Content: Nonauthor Collaborators
*First name, last name, and suffix (if applicable) are required and will appear in PubMed.

***First Name and Middle Initial(s)**	***Last Name**	***Suffix (eg, Jr, III)**	Academic Degrees	Institution	Location (city, state/province, country)	Role or Contribution, eg, chair, principal investigator	Group (if more than 1 Group listed in the byline) and/or Subgroup (eg, Steering Committee)
Jose	Santiago			Puerto Rico Medical Research	USA	Principal Investigator	GKPTN
Shaunak	Dwivedi			MedAccuTrial LLC	USA	Principal Investigator	GKPTN
Steven	Ong			Solano Kidney Care	USA	Principal Investigator	GKPTN
Marwan	Edris			Prime Care Clinical Research	USA	Principal Investigator	GKPTN
Anant	Desai			Renal Consultants Medical Group	USA	Principal Investigator	GKPTN
Marina	Gold			Clearview Medical Research, LLC	USA	Principal Investigator	GKPTN
Bram	Wieskopf			North Georgia Clinical Research	USA	Principal Investigator	GKPTN
Sradha	Kotwal			The George Institute for Global Healt	Australia	Chief Investigator	GKPTN
Hiddo	Lambers Heerspink			The George Institute for Global Healt	Australia	Chief Investigator	GKPTN
Enmoore	Lin			The George Institute for Global Healt	Australia	Project Manager	GKPTN
Sarah	Coggan			The George Institute for Global Healt	Australia	Senior Project Manage	GKPTN
Farjarneh	Hossain			The George Institute for Global Healt	Australia	Senior Clinical Trials As	GKPTN
Shengkun	Sun			George Clinical	China	Clinical Trials Manager	GKPTN
Fan Han	Hsu			George Clinical	Singapore	Program Lead	GKPTN
Emma	Dombroski			George Clinical	New Zealand	Senior Project Manage	GKPTN
Maria	Ali			George Clinical	USA	Chief Medical Officer	GKPTN
Luc	Cambon			George Clinical	Australia	Clinical Research Assoc	GKPTN
Ling	Yap			George Clinical	Malaysia	Clinical Research Assoc	GKPTN
Mai	Ly			George Clinical	Australia	Senior Clinical Researc	GKPTN
Justine	Chua			George Clinical	Malaysia	Clinical Trial Manager	GKPTN
Dominic	Mounsey			George Clinical	Australia	Clinical Trial Specialist	GKPTN
Naomi	Tsukada			George Clinical	Australia	Associate Clinical Trial	GKPTN
Alina	Yoffe			George Clinical	Australia	Senior Clinical Researc	GKPTN
Francisco	Achiaga			George Clinical	Australia	Associate Clinical Trial	GKPTN

Supplemental Online Content: Nonauthor Collaborators
*First name, last name, and suffix (if applicable) are required and will appear in PubMed.

***First Name and Middle Initial(s)**	***Last Name**	***Suffix (eg, Jr, III)**	Academic Degrees	Institution	Location (city, state/province, country)	Role or Contribution, eg, chair, principal investigator	Group (if more than 1 Group listed in the byline) and/or Subgroup (eg, Steering Committee)
Clara	Mok			George Clinical	Australia	Clinical Research Assoc	GKPTN
Emily	Walker			George Clinical	USA	Clinical Trial Specialist	GKPTN
Ann	Reid			George Clinical	UK	Project Manager	GKPTN
Masego	Johnstone			George Clinical	Australia	Clinical Research Assoc	GKPTN
Charles	Czank			George Clinical	Australia	Senior Project Manage	GKPTN
Lisa	Rominger			George Clinical	USA	Project Manager	GKPTN
Paula	Cisternas			George Clinical	Australia	Clinical Research Assoc	GKPTN
Daniel	Rizzi			George Clinical	UK	Clinical Trial Specialist	GKPTN
Joy	Ola			George Clinical	USA	Clinical Research Assoc	GKPTN
David	Garcia			George Clinical	Spain	Clinical Research Assoc	GKPTN
Jessica	Cox			George Clinical	USA	TMF Uploader	GKPTN
Osha	Nelson			George Clinical	USA	TMF Uploader	GKPTN
Lyndal	Hones			George Clinical	Australia	Project Lead	GKPTN
Melinda	Ho			George Clinical	Australia	Global Project Manage	GKPTN
Melissa	Tutt			George Clinical	USA	Clinical Trial Specialist	GKPTN
Fred	Beusenberg			George Clinical	Europe	EU Project Manager	GKPTN
Radhika	Kanade			George Clinical	Australia	EU Regional Scientific I	GKPTN
Eunice	Raymond			George Clinical	Australia	APAC Regional Scientif	GKPTN
Ron	Hamilton			George Clinical	USA	Clinical Research Assoc	GKPTN
Christine	Adeyari			George Clinical	USA	Clinical Research Assoc	GKPTN
Yuehan	Zheng			George Clinical	Japan	Associate Project Man	GKPTN
Joyce	Chow			George Clinical	Hong Kong	Clinical Trial Assistant	GKPTN
Stephanie	Pollard			George Clinical	New Zealand	Senior Clinical Trial Ass	GKPTN
Enrico	Chiari			George Clinical	Europe	Clinical Trial Associate	GKPTN
Olga	Cabrerizo			George Clinical	Spain	Clinical Trial Associate	GKPTN
Denison	Bowman			George Clinical	USA	Clinical Trial Associate	GKPTN
Yuehan	Zheng			George Clinical	China	Clinical Research Assoc	GKPTN
Yiping	Xiao			George Clinical	China	Clinical Research Assoc	GKPTN
Xuejie	Bai			George Clinical	China	Clinical Research Assoc	GKPTN
Joe	Zhou			George Clinical	China	Clinical Research Assoc	GKPTN
Divya	Lokesh			George Clinical	India	Senior Project Analyst	GKPTN

Supplemental Online Content: Nonauthor Collaborators
*First name, last name, and suffix (if applicable) are required and will appear in PubMed.

***First Name and Middle Initial(s)**	***Last Name**	***Suffix (eg, Jr, III)**	Academic Degrees	Institution	Location (city, state/province, country)	Role or Contribution, eg, chair, principal investigator	Group (if more than 1 Group listed in the byline) and/or Subgroup (eg, Steering Committee)
Larry	Larsheid			George Clinical	USA	Senior Project Manage	GKPTN
Naveed	Shabbir			George Clinical	India	Clinical Research Asso	GKPTN
Dana	Hurndon			George Clinical	USA	Senior Clinical Researc	GKPTN
Renee	Garmack			George Clinical	Europe	Project Manager	GKPTN
Liza	Shilpakar			George Clinical	Australia	Clinical Research Asso	GKPTN
Jennifer	Casulla			George Clinical	Australia	Senior Clinical Researc	GKPTN
Hui Ping	Cha			George Clinical	Australia	Senior Clinical Researc	GKPTN
HyeRyun	Jin			George Clinical	Korea	Senior Clinical Researc	GKPTN
Diane	Lickey			George Clinical	USA	Senior Clinical Researc	GKPTN
Jin	Long			George Clinical	China	Clinical Research Asso	GKPTN
Lingling	Bie			George Clinical	China	Clinical Research Asso	GKPTN
Helen	Monaghan			The George Institute for Global Healt	Australia	Platform oversight committee member	CAPTIVATE
Clare	Arnott			The George Institute for Global Healt	Australia	Platform oversight committee member	CAPTIVATE
Gian Luca	di Tanna			University of Applied Sciences and A	Switzerland	Platform oversight committee member	CAPTIVATE
Vicky	Grey			Consumer partner	Australia	Platform oversight committee member	CAPTIVATE
Rathika	Krishnasamy			Sunshine Coast University Hospital,	Australia	Platform oversight committee member	CAPTIVATE
Nicholas	Gray			Sunshine Coast University Hospital,	Australia	MRA DSA committee member	CAPTIVATE
Dean	Guinness			Consumer partner	Australia	CEC	CAPTIVATE
Jeremy	Halewood			Consumer partner	New Zealand	CEC	CAPTIVATE

Supplemental Online Content: Nonauthor Collaborators
*First name, last name, and suffix (if applicable) are required and will appear in PubMed.

***First Name and Middle Initial(s)**	***Last Name**	***Suffix (eg, Jr, III)**	Academic Degrees	Institution	Location (city, state/province, country)	Role or Contribution, eg, chair, principal investigator	Group (if more than 1 Group listed in the byline) and/or Subgroup (eg, Steering Committee)
David	Ioasa			Consumer partner	New Zealand	CEC	CAPTIVATE
Zhangyi	He			The George Institute for Global Health	UK	Project team	CAPTIVATE
Farjaneh	Hossain			The George Institute for Global Health	Australia	project team	CAPTIVATE
Ben	Varley			The George Institute for Global Health	Australia	Project team	CAPTIVATE
Sima	Don			The George Institute for Global Health	Australia	Project team	CAPTIVATE
Nursafwana (Saffy)	Zulkhernain			The George Institute for Global Health	Australia	Project team	CAPTIVATE
Michelle	Kim			The George Institute for Global Health	Australia	Project team	CAPTIVATE
Victoria	Gregory			The George Institute for Global Health	Australia	Project team	CAPTIVATE
