# Supplementary material for: The Global Kidney Patient Trials Network and the CAPTIVATE Platform Clinical Trial Design: A Trial Protocol
Source: JAMA Netw Open. 2024 Dec 11;7(12):e2449998. doi: 10.1001/jamanetworkopen.2024.49998 (PMC11635535; doi:10.1001/jamanetworkopen.2024.49998)
Supplement: Supplement 4. — Data Sharing Statement [file jamanetwopen-e2449998-s004.pdf]

Data Sharing Statement

Kotwal. The Global Kidney Patient Trials Network and the CAPTIVATE Platform Clinical Trial Design. *JAMA Netw Open*. Published December 11, 2024.
doi:10.1001/jamanetworkopen.2024.49998

Data
Data available: Yes
Data types: Deidentified participant data
How to access data: The GKPTN and CAPTIVATE will broadly adhere to the data sharing recommendations and policies of The George Institute for Global Health. De-identified data may be provided to other researchers for future research at the discretion and approval of the trial's Platform Oversight Committee. Assessments of proposals will be based on sound science, benefit-risk balancing and research team expertise. Data will only be provided in a de-identified manner for research in accordance with the local regulatory and ethics approval for the trial
When available: With publication

Supporting Documents
Document types: Other (please specify)
Additional Information: Trial Protocol Data dictionary
How to access documents: Can be requested from the corresponding author
When available: With publication

Additional Information
Who can access the data: Researchers whose proposed use of the data has been approved
Types of analyses: for specified purposes approved by the authors
Mechanisms of data availability: After approval of a proposal, with investigator support and with a signed data access agreement
